# Synthesis and Antimicrobial Activity of Newly Synthesized Nicotinamides

**DOI:** 10.3390/pharmaceutics16081084

**Published:** 2024-08-18

**Authors:** Bojana Anić Marković, Aleksandar Marinković, Jelena Antić Stanković, Stefan Mijatović, Ilija Cvijetić, Milena Simić, Irena Arandjelović

**Affiliations:** 1 Faculty of Technology and Metallurgy, University of Belgrade, Karnegijeva 4, 11000 Belgrade, Serbia; anicbojana89@gmail.com (B.A.M.); marinko@tmf.bg.ac.rs (A.M.); 2 Faculty of Pharmacy, University of Belgrade, Vojvode Stepe 450, 11000 Belgrade, Serbia; milena.simic@pharmacy.bg.ac.rs; 3 Institute of Microbiology and Immunology, Faculty of Medicine, University of Belgrade, Dr. Subotića 1, 11000 Belgrade, Serbia; stefan.mijatovic@med.bg.ac.rs; 4 Faculty of Chemistry, University of Belgrade, Students Square 10-13, 11000 Belgrade, Serbia; ilija@chem.bg.ac.rs

**Keywords:** nicotinamides, nicotinic acid, thiocarbohydrazones, antimicrobial activity

## Abstract

Antioxidants are promising compounds with antimicrobial activity against drug-resistant pathogens, especially when combined with conventional antimicrobials. Our study aimed to characterize the structure of nicotinamides synthesized from nicotinic acid and thiocarbohydrazones and to evaluate their antibacterial and antifungal activity. Seven nicotinic acid hydrazides (NC **1**–**7**) were synthesized using mono-thiocarbohydrazones with hydroxyl group substituents, along with quinolone, phenolic, and pyridine rings known for their antimicrobial activity. The in vitro antimicrobial activity of NC **1**–**7**, at concentrations ranging from 0.001 to 1 mM, was tested against *Staphylococcus aureus* (ATCC 6538), *Enterococcus faecalis* (ATCC 29212), *Pseudomonas aeruginosa* (ATCC 27853), *Klebsiella pneumoniae* (NCIMB 9111), and *Candida albicans* (ATCC 24433) using the broth microdilution method per EUCAST 2024 guidelines. Microorganism survival percentages were calculated based on optical density, and target fishing using the PharmMapper database identified potential molecular targets. The results showed that *P. aeruginosa* was most susceptible to the compounds, while *C. albicans* was the least susceptible. NC **3** significantly inhibited *P. aeruginosa* and *K. pneumoniae* growth at 0.016 mM, while higher concentrations were required for *S. aureus*, *E. faecalis*, and *C. albicans*. NC **5** was most effective against gram-positive bacteria at 0.03 mM. Only NC **4** completely inhibited *C. albicans* below 1 mM. NC **3**, with the lowest concentration for 50% growth inhibition (0.016–0.064 mM), showed promising antibacterial potential against specific AMR-related proteins (bleomycin resistance protein, HTH-type transcriptional regulator QacR, and streptogramin A acetyltransferase), suggesting that this class of compounds could enhance or restore the activity of established antibiotics.

## 1. Introduction

With an estimated five million deaths annually worldwide, antimicrobial resistance (AMR) has become a hallmark of global public health threats [1]. So far, resistance has been reported to all antimicrobials currently used in human medicine, while only a few novel drugs are in the pipeline. According to the latest report of the European Center for Disease Prevention and Control, the percentage of invasive bacterial isolates with resistance to last-line antibiotics in the European region has been increasing from 2017 to 2021. The constant rise in carbapenem-resistant *Klebsiella pneumoniae* and *Acinetobacter* spp., as well as vancomycin-resistant *Enterococcus* spp. in previous years, underscores the pressing concern of AMR in Europe [2] and confirms the urgent need for the design and synthesis of new active molecules.

Within the current landscape of AMR, there is a swift emergence of antifungal resistance, adding another layer of complexity to the global health challenge presented by invasive fungal diseases (IFD) [3]. The increasing trend of IFD is largely influenced by advancements in modern medicine and the widespread availability of treatments that compromise the immune system. Additionally, new at-risk groups for IFD, such as individuals with chronic obstructive pulmonary disease, liver or kidney disease, viral respiratory tract infections like influenza, and those with previous non-tuberculous mycobacterial infections, are consistently being identified. Moreover, the COVID-19 pandemic has intensified the issue, with frequent reporting of comorbid invasive candidiasis, aspergillosis, and mucormycosis, often resulting in treatment failure [4,5]. In 2022, the World Health Organization (WHO) published the first-ever list of fungal priority pathogens, which, together with the bacterial priority pathogens list published in 2017, aims to stimulate global action on the research and development of new antimicrobials [6].

The fight against drug-resistant pathogens is advancing with research into alternative antimicrobial strategies. Among these, plant extracts, metal ions, and nanoparticles show great potential. Natural compounds from plants possess unique mechanisms to combat resistant pathogens, often reducing the likelihood of resistance development. For instance, *Lavandula angustifolia* essential oil demonstrated significant antifungal activity against *C. albicans* sputum isolates [7]. Also, secondary metabolites from plants like *Dioscorea altissima*, *Olea europaea*, *Thymus lamiaceae,* and *Origanum vulgare* have shown remarkable antimicrobial effects on bacteria and fungi responsible for oral and dental diseases [8]. In recent years, the development of metalloantimicrobials, including metal ions and nanoparticles, has introduced new therapeutic options for drug-resistant bacteria and fungi. For instance, a newly synthesized aryloxycyclotriphosphazene compound, used in a silver-containing gel for wound healing, inhibited wound infection agents like *Staphylococcus aureus, Pseudomonas aeruginosa,* and *C. albicans* [9]. Metal-based nanoparticles exhibit ideal physiochemical performances for antimicrobial activity, such as enhancing drug delivery to infectious sites, maintaining long-term blood concentrations, controlling drug release, and potentially reversing drug resistance [10,11].

In addition to advanced breakthroughs in biotechnology, recent developments in synthetic chemistry offer significant opportunities for researching innovative therapies to combat AMR. Recent findings have highlighted the potential role of antioxidants as promising antimicrobial agents against drug-resistant pathogens, particularly when combined with conventional antimicrobials [12]. The antimicrobial effect of antioxidants is thought to arise from several mechanisms, including boosting cell wall permeability, regulating redox reactions, inhibiting adhesion molecule expression, and decreasing the production of proinflammatory cytokines [13]. Newly synthesized compounds based on nicotinamide, the active form of vitamin B3, are of particular importance due to their documented antibacterial [13,14,15,16] and antifungal [17,18,19] activities, attributed to the presence of thiol and phenol groups in their structure. Moreover, Tcherniuk et al. showed that a high concentration of nicotinamide effectively inhibits the growth of blood-stage *Plasmodium falciparum* and works synergistically with artemisinin, chloroquine, and pyrimethamine without causing erythrocyte toxicity [20]. This suggests that nicotinamide may also potentiate the antiparasitic effects of common antimalarial drugs. Additionally, Zhou et al.’s findings provide preliminary evidence supporting the safety and therapeutic efficacy of nicotinamide in treating *Leishmania* infection in BALB/c mice, highlighting its potential as a treatment for visceral leishmaniasis [21].

The effects of nicotinamide are primarily driven by an increase in the levels of nicotinamide adenine dinucleotide (NAD^+^), an essential molecule from a cellular functional standpoint [22,23]. NAD^+^ supplementation has been shown to boost the presence of anti-inflammatory macrophages, minimize neurodegeneration, activate autophagy and mitophagy processes, and uphold genome stability, consequently reducing inflammation, diminishing tissue atrophy, improving cognitive function, and enhancing insulin sensitivity [22,24,25]. However, although the effectiveness of nicotinamide in preventing the onset of diabetes mellitus has still remained uncertain [26,27], this compound is considered progressively promising in the prevention of ischemic reperfusion in nerve and vascular cells [28], neurological dysfunctions [29,30,31,32], and development of distinct psychological disorders [33]. Several studies also implied the prosperous use of nicotinamide against the development of uremic pruritus, inflammatory diseases [34], photo-aging, skin cancers [35], and even HIV reproduction [36,37]. Nevertheless, it is used for the treatment and prevention of skin disorders, such as pellagra [38], bullous pemphigoid [39], and acne lesions [40], and as an effective ingredient in various cosmetic products as well. Nicotinamide is considered a safe and well-tolerated compound with minor side effects, including nausea, vomiting, headache, and fatigue when administered orally in doses of 1–3 g/day [41].

The aim of our study was twofold: (1) to perform chemical characterization of nicotinamides synthesized from nicotinic acid and thiocarbohydrazones and (2) to determine the antimicrobial activity of newly synthesized nicotinamides against strains of selected bacteria and fungi prioritized by the WHO to guide discovery, research, and development of new antibiotics for drug-resistant infections, and which are among the most frequently isolated resistant strains in Serbia [2,6]. Nicotinic acid hydrazides were synthesized by linking two pharmacophores through a condensation reaction [42]. Mono-thiocarbohydrazones with hydroxyl group substituents, as well as quinoline, phenolic, and pyridine rings, were selected for this process due to their demonstrated antimicrobial activity [43].

## 2. Materials and Methods

### 2.1. Materials

Nicotinamide compounds (NC) **1**–**7** were synthesized by using thionyl chloride (≥99%), nicotinic acid (pyridine-3-carboxylic acid) (≥98%) (Fluka AG–Chemie, Buchs, Switzerland), benzaldehyde (98%) (CDH chemicals, New Delhi, India), 2-quinolinecarboxaldehyde (≥98), salicylaldehyde (98%), 2-pyridinecarboxaldehyde (≥99%), 2-acetylpyridine (≥99%) (Sigma Aldrich, Buchs, Switzerand), 8-quinolinecarboxaldehyde (98%), 8-hydroxy-2 quinolinecarboxaldehyde (98%) (Thermo Fisher Scientific, Waltham, MA, USA), dimethylformamide (DMF) (99%), 1-Ethyl-3-(3-dimethylaminopropyl)carbodiimide hydrochloride (EDC∙HCl), hydroxybenzotriazole (HOBt), dichloromethane (CH_2_Cl_2_), tetrahydrofuran (THF), triethylamine (Et_3_N), and dimethyl sulfoxide (DMSO) (99%) (Merck KGaA, Darmstadt, Germany).

### 2.2. Characterization of Synthesized Compounds

All compounds were characterized by using the following spectroscopic methods: Fourier Transform Infrared Spectroscopy (FTIR), elemental analysis, mass spectrometry, as well as ^1^H and ^13^C, and Heteronuclear Single Quantum Coherence (HSQC) nuclear magnetic resonance (NMR) spectroscopy. Elemental analysis (C, H, N) was performed using a VARIO EL III elemental analyzer (Elementar, Langelselbold, Germany). The ATR-FTIR spectra were recorded on a Thermo Scientific^TM^ Nicolet^TM^ iS^TM^ 10 spectrometer (Thermo Fisher Scientific, Waltham, MA, USA) with a smart iTX^TM^ accessory. The NMR spectra were recorded on Bruker Ascend 400 (400 MHz) spectrometer (Bruker, Billerica. MA. USA). Chemical shifts are given in parts per million (δ) downfield from tetramethylsilane as the internal standard. For the mass spectrometric (MS) analysis, a linear ion trap mass spectrometer LTQ XL (Thermo Fisher Scientific, Waltham, MA, USA) was used. The MS analysis was performed using the electrospray ionization (ESI) technique in the negative mode. The optimal ESI parameters were as follows: capillary temperature (220 °C), capillary voltage (−24.5 kV), sheath gas (9 au, i.e., 9 arbitrary units), and auxiliary gas (23 au). The mass spectra were recorded in the range of *m*/*z* 50−500. Solutions of synthesized nicotinamides were prepared in methanol at a concentration of 10 µg mL^−1^.

FTIR, NMR, and MS spectra for all compounds are given in the Supplementary Materials.

### 2.3. General Procedure for the Preparation of Nicotinamides (NC ***1**–**7***)

Two synthesis methods were used in this research. The first method included the synthesis of nicotinoyl chloride from nicotinic acid and SOCl_2_, followed by condensation with *mono*-thiocarbohydrazone to yield nicotinamides. This method was applied for the synthesis of NC **2**–NC **5** and NC **7** nicotinamides. The second method involved the synthesis of nicotinamides from the same starting materials but using EDC∙HCl as a coupling agent and HOBt as a carboxylic group activator. Using Method II, compounds NC **1** and NC **6** were synthesized. The apparatus was thoroughly dried before synthesis, and a reflux condenser was protected with a calcium chloride tube. More details about both methods are provided in the following paragraphs.

**Scheme 1:**
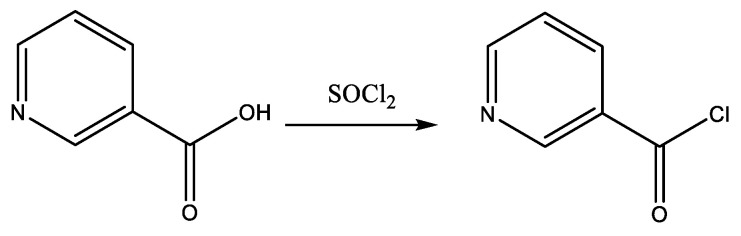
Synthesis of nicotinoyl chloride.

**Scheme 2:**
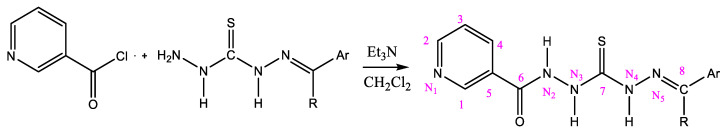
Synthesis of nicotinamides by amidation of nicotinoyl chloride with *mono*-thiocarbohydrazones.

Method I:

Nicotinic acid (5 mmol) was dissolved in 50 mL of THF in a 100 mL three-necked round-bottom flask. SOCl_2_ (20 mmol) was added dropwise over 20 min using a dropping funnel. The reaction mixture was then heated under reflux for 3 h (Scheme 1). After the end of gaseous product liberation, the excess SOCl_2_ was removed by distillation. The resulting nicotinoyl chloride, obtained as the distillation residue, was dissolved in 30 mL of dry CH_2_Cl_2_. The solution was externally cooled to 0 °C using an ice bath, and a mixture of *mono*-thiocarbohydrazone (5 mmol) and triethylamine (5 mmol) in 10 mL of dry CH_2_Cl_2_ was added dropwise over 30 min. Triethylamine (Et_3_N) was used as an acid-trapping agent. The reaction mixture was then stirred for 3 h at room temperature and 1 h at 40 °C. The resulting product was filtered, rinsed with ice water, and crystallized from ethanol (Scheme 2).

**Scheme 3:**
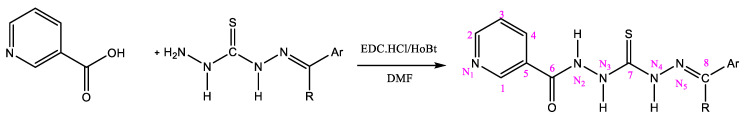
Synthesis of nicotinamide using a direct coupling with EDC.

Method II:

Nicotinic acid (5 mmol) was dissolved in 10 mL of DMF in a 100 mL two-necked round-bottom flask immersed in an ice bath. HOBt (5 mmol), dissolved in 5 mL DMF, was added after the acid was dissolved. The *mono*-thiocarbohydrazone (5 mmol), dissolved in 15 mL of DMF, was added dropwise over 10 min, followed by the addition of EDC∙HCl solution (5 mmol in 10 mL of DMF). The reaction mixture was stirred for 5 h at room temperature and then poured into ice water to crystallize (Scheme 3). The product was washed with ice water (50 mL) and crystallized from ethanol.

### 2.4. Antimicrobial Susceptibility Testing by Resazurin Assay

NC **1**–**7** compounds, as well as levofloxacin, amikacin, and fluconazole (Sigma–Aldrich, Taufkirchen, Germany) powders, were dissolved in 100% dimethyl sulfoxide (DMSO, Merck, Darmstadt, Germany) at final concentrations 200 mM, 1.6 mg/mL, 25.6 mg/mL, and 12.8 mg/mL, respectively. Prepared stock solutions were stored at −70 °C until use.

The in vitro antimicrobial activity was tested against *Staphylococcus aureus* (ATCC 6538), *Enterococcus faecalis* (ATCC 29212), *Pseudomonas aeruginosa* (ATCC 27853), *Klebsiella pneumoniae* (NCIMB 9111), and *Candida albicans* (ATCC 24433) by broth microdilution method proposed by the European Committee on Antimicrobial Susceptibility Testing [44] with some modifications. In brief, suspensions of tested microorganisms were prepared from overnight cultures by suspending them in 0.85% saline at a density of 0.5 McFarland (Bio-Merieux, Marcy-l’Étoile, France) and further diluted 1:10 in Müeller-Hinton broth (MHB, Torlak, Serbia) and Sabouraud dextrose broth (SDB, Torlak, Serbia) to a final concentration of 1–5 × 10^5^ CFU/mL. Stock solutions of NC **1**–**7** and antimicrobial compounds were dissolved at a 1:100 ratio in MHB and SDB, followed by 10 serial two-fold dilutions. A total of 100 µL preformed dilutions of each compound were added to the flat-bottomed 96-well microtiter plates. After adding 100 µL of work suspensions to appropriate wells, the range concentrations of NC **1**–**7**, levofloxacin, amikacin, and fluconazole were 0.001–1 mM, 8–0.016 µg/mL, 128–0.25 µg/mL, and 64–0.06 µg/mL, respectively. The final concentration of DMSO in the cultures did not exceed 0.5%. Compounds-free and sterility-control wells were included as well. A nutrition medium with the corresponding concentrations of tested compounds was used as a blank. All microtiter plates were incubated at 37 °C for 24 h in an aerobic atmosphere.

After incubation, the plates were estimated to have microbial growth. The minimum inhibitory concentration (MIC) of each compound was defined as the lowest concentration at which no color change of resazurin occurs, indicating complete growth inhibition. All tests were performed in triplicates. Results were reported as modal values.

### 2.5. Microbe Growth Curve Analysis

Optical density (OD) measurement was used to determine the growth of microorganisms [45] in the presence of NC **1**–**7**, as described in Section 2.2. The OD was measured at 492 nm using a Multiskan™ FC Microplate Photometer (Thermo Fisher Scientific Inc.; Waltham, MA, USA) after incubation at 37 °C for 24 h for bacteria and 48 h at 37 °C for *C. albicans*. The percentage of survival of microorganisms (S) in culture was calculated according to the formula:

S (%) = [(OD_sample_ − OD_blank_)/(OD_positive control_ − OD_blank_)] × 100,

where OD_positive control_ represents the OD of the non-treated growth control, while OD_sample_ indicates the OD of the tested compound, and OD_blank_ refers to the OD of the appropriate blank. The percentage of microbial growth inhibition was calculated as %dead = 100 − S. The inhibitory concentration required to inhibit the growth of 50% and 90% of microorganism cells are expressed as MIC_50_ and MIC_90_, respectively. All measurements were applied in triplicate two times, and the results were reported as mean values.

### 2.6. Target Fishing

We performed target fishing in order to reveal potential antimicrobial molecular targets using the PharmMapper online database [46,47]. Starting with the 3D structure of NC **3**, optimized by the semiempirical PM7 method [48] in MOPAC2016, we generated 100 conformers using Cyndi [49]. The NC **3** was chosen as the model compound for target fishing due to its highest potency, as indicated by its MIC_50_ and MIC_90_ values against all examined microbial strains (Table 1). The pharmacophores of these conformers were then screened against a database of 7302 molecular targets from the Protein Databank (PDB). Employing a genetic algorithm (GA), we enhanced the accuracy of the alignment between ligand and protein pharmacophores. The excluded volumes were also incorporated to restrict the overlap within the active site volume. Subsequently, the best 300 solutions were exported and analyzed for bacterial proteins. Solutions were ranked according to *z*-score, and only those with *z* > 2 were considered for further molecular docking studies.

AutoDock Vina 1.1. [50] was utilized to dock the NC **3** into the active sites of the three most relevant bacterial targets identified through pharmacophore search: bleomycin resistance protein (PDB code 1EWJ), HTH-type transcriptional regulator QacR (1JT6), and streptogramin A acetyltransferase (1KHR). Additionally, dihydrofolate reductase (DHFR) was suggested as a potential target based on ChemBL search, and DHFR from *M. tuberculosis* (1DG5) was utilized for molecular docking. The protein structures for docking were prepared by removing crystallized water molecules, ions, and co-crystallized ligands to create space for ligand docking. All residues within a 12 Å radius around the co-crystallized ligand were selected for active site definition. The ionization state was set to resemble pH 7.4 using PROPKA [51]. The exhaustiveness was set to 250, and 20 solutions were retained for each target. All calculations were executed using Vega ZZ 3.2.3 as a graphical user interface [52]. The ADME properties and drug-likeness of NC **3** were predicted using the SwissADME web server [53].

### 2.7. Statistical Analysis

The normality distribution of the variables was verified using the Kolmogorov–Smirnov and Shapiro–Wilk tests. MIC values obtained by resazurin and OD measurement methods were compared by using the Mann–Whitney U test. Kruskal–Wallis test with post hoc pairwise comparison (Mann-Whitney U test) and applied Bonferroni correction were used to compare MIC_50_ values of the same compound on different strains. In all the cases, *p* values < 0.05 were considered as statistically significant. All statistical analyses were performed using Excel (version 16; Microsoft Corp., Redmond, WA, USA) for Windows 10 (Microsoft Corp., Redmond, WA, USA) and R (R package ver. 2.7-1; R Core Team, Vienna, Austria).

## 3. Results and Discussion

### 3.1. Chemistry

In this study, we used two methods to synthesize nicotinamides from nicotinic acid and various *mono*-thiocarbohydrazones. The first method includes activation of acid with thionyl chloride (Scheme 1), followed by amidation with *mono*-thiocarbohydrazones (Scheme 2). The second method is a direct, one-step coupling using the well-known EDC as a coupling agent (Scheme 3).

#### 3.1.1. (NC **1**) 1-[(2-Hydroxyphenyl) methyl]-5-(pyridine-3-carbonyl) Dihydrazide Thiocarbonyl Acid

After synthesis, a slightly yellow powder was obtained with a yield of 72% and a melting point of 48.9 °C.

Elem. anal.: Measured on C_14_H_13_N_5_O_2_S (Mw = 315.35 gmol^−1^): C, 53.32; H, 4.16; N, 22.21; O, 10.15; S, 10.17%. Found: C, 53.36; H, 4.14; N, 22.28; O, 10.08; S, 10.17%. IR(KBr, cm^−1^) νmax: 3054.48 (N-H vibration stretching of amide group), 1686.08 (C=O vibration stretching of amide group), 1489.52 (C=S vibration), 1583.23 (C=N vibration).^1^H NMR (400 MHz, DMSO-d6, δ(ppm)): 6.84–6.91 (m, 2H, H-C_13_, H-C_11_); 7.25 (t, 1H, J = 8.0 Hz, H-C_12_); 7.58 (s, 1H, H-C_3_); 8.08 (d, 1H, J = 8.0 Hz, H-C_14_); 8.26 (d, 1H, J = 8.0 Hz, H-C_4_), 8.45 (s, 1H, H-C_2_); 8.78 (d, 1H, J = 4.0 Hz, H-C_8_); 9.08 (s, 1H, H-C_1_), 9.99 (brs, 1H, H-N_3_); 10.35 (brs,1H, H-N_2_); 10.78 (s, 1H, H-C_10_(OH)), 11.84 (s, 1H, H-N_4_). ^13^C NMR (100 MHz, DMSO-d6) δ ppm, TMS): 116.58 (C_11_); 119.69 (C_9_); 120.66 (C_13_); 124.11 (C_3_); 127.49 (C_14_); 129.16 (C_5_); 131.81 (C_12_); 135.74 (C_4_); 141.03 (C_8_); 148.97 (C_2_); 152.85 (C_1_); 157.07 (C_10_); 164.78 (C=O); 179.43 (C=S), [M−H]^−^, *m*/*z* 314.28.

#### 3.1.2. (NC **2**) 1-(2-Pyridinylmethylene)-5-(pyridine-3-carbonyl) Dihydrazide Thiocarbonyl Acid

After synthesis, a slightly yellow powder was obtained with a yield of 52% and a melting point of 207.6 °C.

Elem. anal.: Measured on C_13_H_12_N_6_OS (Mw = 300.34 gmol^−1^): C, 51.99; H, 4.03; N, 27.98; O, 5.33; S, 10.67%. Found: C, 52.00; H, 4.02; N, 27.91; O, 5.43; S, 10.64%. IR (KBr, cm^−1^) νmax: 3145.36 (N-H vibration stretching of amide group), 1669.80 (C=O vibration stretching of amide group), 1470.97 (C=S vibration), 1590.27 (C=N vibration). ^1^H NMR (400 MHz, DMSO-d6) δ(ppm)): 7.38–7.42 (m, 1H, H-C_8_); 7.58–7.59 (m, 1H, H-C_12_); 7.86–7.90 (m, 1H, H-C_3_), 8.08–8.44 (m, 3H, H-C_11_, C_10_, H-C_4_); 8.56–8.60 (m, 1H, H-C_13_); 8.74–8.78 (m, 1H, H-C_2_); 9.10 (s, 1H, H-C_1_), 10.86 (s, 1H, H-N_3_), 11.80 (s,1H, H-N_2_), 12.14 (s, 1H, H-N_4_). ^13^C NMR (100 MHz, DMSO-d6, δ ppm, TMS): 121.04 (C_10_); 124.19 (C_3_); 124.81 (C_12_); 129.10 (C_5_); 135.80 (C_4_); 137.15 (C_11_); 144.00 (C_1_); 148.93 (C_2_); 149.80 (C_13_); 152.90 (C_8_); 153.59 (C_9_); 164.80 (C=O); 179.94 (C=S), [M−H]^−^, *m*/*z* 299.17.

#### 3.1.3. (NC **3**) 1-[1-(2-Pyridinyl)ethylidene]-5-(pyridine-3-carbonyl) Dihydrazide Thiocarbonyl Acid

After synthesis, a yellow powder was obtained with a yield of 84% and a melting point of 200.5 °C.

Elem. anal.: Measured on C_14_H_14_N_6_OS (Mw = 314.37 gmol^−1^): C, 53.49; H, 4.49; N, 26.73; O, 5.09; S, 10.20%. Found: C, 53.45; H, 4.54; N, 26.74; O, 5.08; S, 10.20%. IR (KBr, cm^−1^) νmax: 3181.03 (N-H vibration stretching of amide group), 1636.07 (C=O vibration stretching of amide group), 1466.72 (C=S vibration), 1596.43 (C=N vibration).^1^H NMR (400 MHz, DMSO-d6, δ(ppm)): 2.45 (d, 3H, J = 24.0 Hz, H-C_9_); 7.42 (t, 1H, J = 4.0 Hz, H-C_3_), 7.58 (t, 1H, J = 4.0 Hz, H-C_13_); 7.83 (t, 1H, J = 8.0 Hz, H-C_12_); 8.28 (d, 1H, J = 8.0 Hz, H-C_11_); 8.59–8.61 (m, 2H, H-C_4_,H-C_14_); 8.79 (d, 1H, J = 4.0 Hz, H-C_2_); 9.10 (s, 1H, H-C_1_); 10.48 (s, 1H-N_4_); 10.86 (s, 1H, H-N_3_); 10.88 (s, 1H, H-N_2_). ^13^C NMR (100 MHz, DMSO-d6, δ ppm, TMS): 12.71 (C_9_); 121.70 (C_12_); 124.14 (C_3_); 124.61 (C_10_); 129.15 (C_5_); 135.76 (C_4_); 136.78 (C_11_); 148.96 (C_1_, C_2_); 150.11 (C_14_); 152.87 (C_13_); 154.93 (C_8_); 164.71 (C=O); 180.62 (C=S), [M−H]^−^, *m*/*z* 313.28.

#### 3.1.4. (NC **4**) 1-(8-Quinolylmethylene)-5-(pyridine-3-carbonyl) Dyhidrazide Thiocarbonyl Acid

After synthesis, a brown powder was obtained with a yield of 73% and a melting point of 191.8 °C.

Elem. anal.: Measured on C_17_H_14_N_6_OS (Mw = 350.40 gmol^−1^): C, 58.27; H, 4.03; N, 23.98; O, 4.57; S, 9.15%. Found: C, 58.21; H, 4.11; N, 23.96; O, 4.60; S, 9.12%.

IR (KBr, cm^−1^) νmax: 3167.12 (N-H vibration stretching of amide group), 1667.82 (C=O vibration stretching of amide group), 1473.60 (C=S vibration), 1592.34 (C=N vibration). ^1^H NMR (400 MHz, DMSO-d6, δ(ppm)): 7.54–7.63 (m, 2H, H-C_3_, H-C_14_), 7.70 (t, 1H, J = 8.0 Hz, H-C_11_); 8.08 (d, 1H, J = 4.0 Hz, H-C_4_); 8.27 (d, 1H, J = 8.0 Hz, H-N_10_); 8.44 (d, 1H, J = 8.0 Hz, H-C_12_); 8.70–8.81 (m, 2H, H-C_8_, H-C_13_); 8.99 (s, 1H, H-C_2_); 9.06 (s, 1H, H-C_15_); 9.42 (s, 1H, H-C_1_); 10.56 (s, 1H, H-N_3_); 10.83 (s, 1H, H-N_2_); 12.15 (s, 1H, H-N_4_). ^13^C NMR (100 MHz, DMSO-d6, δ ppm, TMS): 122.31 (C_11_); 124.15 (C_14_); 126.89 (C_3_, C_12a_); 128.46 (C_10_); 129.16 (C_12_); 130.53 (C_5_); 131.38 (C_9_); 135.76 (C_4_); 137.12 (C_13_); 140.74 (C_8_); 145.89 (C_15a_); 148.97 (C_2_); 150.92 (C_1_); 152.87 (C_15_); 164.82 (C=O); 179.76 (C=S), [M−H]^−^, *m*/*z* 349.17.

#### 3.1.5. (NC **5**) 1-(2-Quinolylmethylene)-5-(pyridine-3-carbonyl) Dihydrazide Thiocarbonyl Acid

After synthesis, a slightly yellow powder was obtained with a yield of 53% and a melting point of 174.7 °C.

Elem. anal.: Measure on C_17_H_14_N_6_OS (Mw = 350.40 gmol^−1^): C, 58.27; H, 4.03; N, 23.98; O, 4.57; S, 9.15%. Found: C, 58.20; H, 4.10; N, 23.93; O, 4.65; S, 9.12%. IR (KBr, cm^−1^) νmax: 3191.56 (N-H vibration stretching of amide group), 1668.0 (C=O vibration stretching of amide group), 1501.50 (C=S vibration), 1596.50 (C=N vibration). ^1^H NMR (400 MHz, DMSO-d6, δ(ppm)): 7.44–7.66 (m, 2H, H-C_3_, H-C_13_); 7.80 (t, 1H, J = 8.0 Hz, H-C_8_); 8.02–8.06 (m, 2H, H-C_10_, H-C_14_); 8.30–8.31 (m, 2H, H-C_12_, H-C_15_); 8.42 (d, 1H, J = 6.0 Hz, H-C_4_), 8.61 (dd, 1H, H-C_11_); 8.79 (d, 1H, J = 4.0 Hz, H-C_2_), 9.12 (s, 1H, H-C_1_); 10.75 (s, 1H, H-N_3_), 10.91 (s, 1H, H-N_2_); 12.30 (s, 1H, H-N_4_). ^13^C NMR (100 MHz, DMSO-d6, δ ppm, TMS): 118.77 (C_10_); 124.18 (C_3_); 127.74 (C_13_); 128.39 (C_12_, C_15_); 129.31 (C_5_, C_14_); 130.45 (C_11a_); 135.75 (C_4_); 136.79 (C_11_); 144.14 (C_8_); 147.84 (C_2_); 148.96 (C_1_); 152.94 (C_9_, C_15a_); 164.81 (C=O), 179.95 (C=S), [M−H]^−^, *m*/*z* 349.32.

#### 3.1.6. (NC **6**) 1-[(8-Hydroxy)-2-quinolylmethylene]-5-(pyridine-3-carbonyl) Dihydrazide Thiocarbonyl Acid

After synthesis, a brown powder was obtained with a yield of 81% and a melting point of 210 °C.

Elem. anal.: Measured on C_17_H_14_N_6_O_2_S (Mw = 366.40 gmol^−1^): C, 55.73; H, 3.85; N, 22.94; O, 8.73; S, 8.75%. Found: C, 55.80; H, 3.78; N, 22.99; O, 8.68; S, 8.75%. IR (KBr, cm^−1^) νmax: 3200.65 (N-H vibration stretching of amide group), 1658.31 (C=O vibration stretching of amide group), 1455.72 (C=S vibration), 1590.32 (C=N vibration).

^1^H NMR (400 MHz, DMSO-d6, δ(ppm)): 7.13 (d, 1H, J = 8.0 Hz, H-C_14_); 7.42–7.46 (m, 2H, H-C_3_, H-C_8_); 7.58–7.62 (m, 1H, H-C_13_); 8.33–8.41 (m, 3H, H-C_4,_ H-C_10_, H-C_12_); 8.57 (d, 1H, J = 8.0 Hz, H-C_11_); 8.80 (d, 1H, J = 4.0 Hz, H-C_2_); 9.12 (s, 1H, H-C_1_) 9.90 (s, 1H, H-C_15_(OH)), 10.72 (s, 1H, H-N_2_), 10.91 (s, 1H, H-N_3_); 12.35 (s, 1H, H-N_4_).

^13^C NMR (100 MHz, DMSO-d6, δ ppm, TMS): 112.62 (C_14_); 118.23 (C_12_); 119.09 (C_10_); 124.18 (C_3_); 128.70 (C_13_); 129.09 (C_5_); 129.34 (C_11a_); 135.76 (C_4_); 136.64 (C_11_); 138.68 (C_15a_); 144.00 (C_8_); 148.96 (C_2_); 152.09 (C_1_); 152.93 (C_9_), 153.93 (C_15_); 164.83 (C=O); 179.95 (C=S), [M−H]^−^, *m*/*z* 365.37, [M-OH]^−^, *m*/*z* 349.30.

#### 3.1.7. (NC **7**) 1-(Phenyl-methylen)-5-(pyridine-3-carbonyl) Dihydrazide Thiocarbonyl Acid

After synthesis, a white powder was obtained with a yield of 64% and a melting point of 191 °C.

Elem. anal.: Measured on C_14_H_13_N_5_OS (Mw = 299.35 gmol^−1^): C, 56.17; H, 4.38; N, 23.40; O, 5.34; S, 10.71%Found: C, 56.20; H, 4.47; N, 23.63; O, 5.35; S, 10.35%. IR (KBr, cm^−1^) νmax: 3211.24 (N-H vibration stretching of amide group), 1660.20 (C=O vibration stretching of amide group), 1449.19 (C=S vibration), 1600.27 (C=N vibration).

^1^H NMR (400 MHz, DMSO-d6, δ(ppm)): 7.44 (s, 3H, H-C_11_, H-C_12_, H-C_13_); 7.58 (t, 1H, J = 4.0 Hz, H-C_3_); 7.91 (s, 1H, H-C_10_); 7.92 (s, 1H, H-C_14_); 8.13 (s, 1H, H-C_4_); 8.27 (d, 1H, J = 8.0 Hz, H-C_8_); 8.78 (s, 1H, H-C_2_); 9.09 (s, 1H, H-C_1_); 10.44 (s, 1H, H-N_3_); 10.81 (s, 1H, H-N_2_); 11.93 (s, 1H, H-N_4_).

^13^C NMR (100 MHz, DMSO-d6, δ ppm, TMS): 124.15 (C_3_); 128.07 (C_11_, C_13_); 129.14 (C_10_, C_14_, C_5_); 130.54 (C_12_); 134.49 (C_9_); 135.76 (C_4_); 143.88 (C_8_); 148.96 (C_2_); 152.86 (C_1_); 164.80 (C=O); 179.69 (C=S), [M−H]^−^, *m*/*z* 298.17.

IR spectrophotometry showed characteristic peaks of amide bond, thiol form, and carbonyl group. Stretching vibrations of the amide group are presented around 3054–3211 cm^−1^. Characteristic strong absorption bands between 1583 and 1600 cm^−1^ are attributed to the azomethin ν(C=N) stretching vibration. The stretching band of amide group ν(C=O) shows absorption between 1636 and 1686 cm^−1^. Group characteristics for thiocarbohydrazide are shown between 1449 and 1501 cm^−1^ as the form ν(C=S) and around 2600–2800 cm^−1^ for ν(S=H) band.

^1^HNMR spectroscopy showed characteristic peaks of nicotinic amides for N-H hydrogens according to δ 10.48–12.30 for N_4_ atom, δ 9.99–10.87 for N_3_ atom and δ 10.35–11.8 for N_2_ atom. Near them is a singlet for O-H atom on δ 9.88–10.78 for NC **1** and NC **6**. Also, one of the characteristic peaks is azomethine (-CH=N), which could be seen between δ 7.40 and 8.79 with the exception of NC **3**. NC **3** has a methyl group (-CH_3_) connected to azomethine moiety, giving a singlet on δ 2.45. Aromatic atoms from nicotinic acid, phenol, and quinole nuclei are shown between δ 6.87 and 9.42.

^13^CNMR spectrum showed characteristic peaks for all seven compounds. Thione carbon (C=S) is present between 179.43 and 180.62 ppm, carbonyl carbon (C=O) is shown at 164.71–164.83 ppm, and azomethine carbon (C=N) between 140.74 and 154.93 ppm. Compound **3** has a characteristic methyl group at 12.71 ppm. The carbonyl atom attached to the O-H group at NC **1** and NC **6** is from 127.49 to 153.93 ppm. Also, carbonyl atoms resulting from phenyl and quinoline groups are shown between 112.62 and 157.07 ppm.

The MS analysis was used for the identification of analytes present in the synthesized nicotinamides. A mass spectrum was obtained for each sample. Mass spectra have shown that masses of the obtained analytes correspond to deprotonated [M−H]^−^ ions of nicotinamide derivatives, and those ions were the most intensive in the obtained spectra.

### 3.2. Minimum Inhibitory Concentrations of NC ***1**–**7***

The resazurin reduction method is frequently employed to evaluate the active growth of bacterial and fungal cells by directly correlating with viable cell quantity. Nevertheless, while this method allows for the estimation of complete growth inhibition, it does not facilitate the estimation of microbial survival percentage. While studies have been conducted assessing the percentage of survival of treated cells based on absorbance values, the results of resazurin viability assay are influenced by various factors, such as dye concentration, incubation time, and reading results at different wavelengths due to the appearance of different shades of red and purple. Consequently, this test cannot be considered reliable in determining the percentage of growth inhibition of microorganism-treated cells [54]. However, no differences were observed in our investigation between inhibitory concentrations obtained by the resazurin method and MIC_90_ values for the tested strains (*p* = 0.363).

Based on the resazurin viability test results, none of the tested compounds completely inhibited the growth of *S. aureus* and *E. faecalis* (Table 2). However, regarding gram-negative strains *K. pneumoniae* and *P. aeruginosa*, NC **3** resulted in growth inhibition at a lower concentration (0.032 mM). For *C. albicans*, only NC **4** in concentration lower than 1 mM exhibited complete growth inhibition.

### 3.3. Growth Curve Analysis

**Figure 1:**
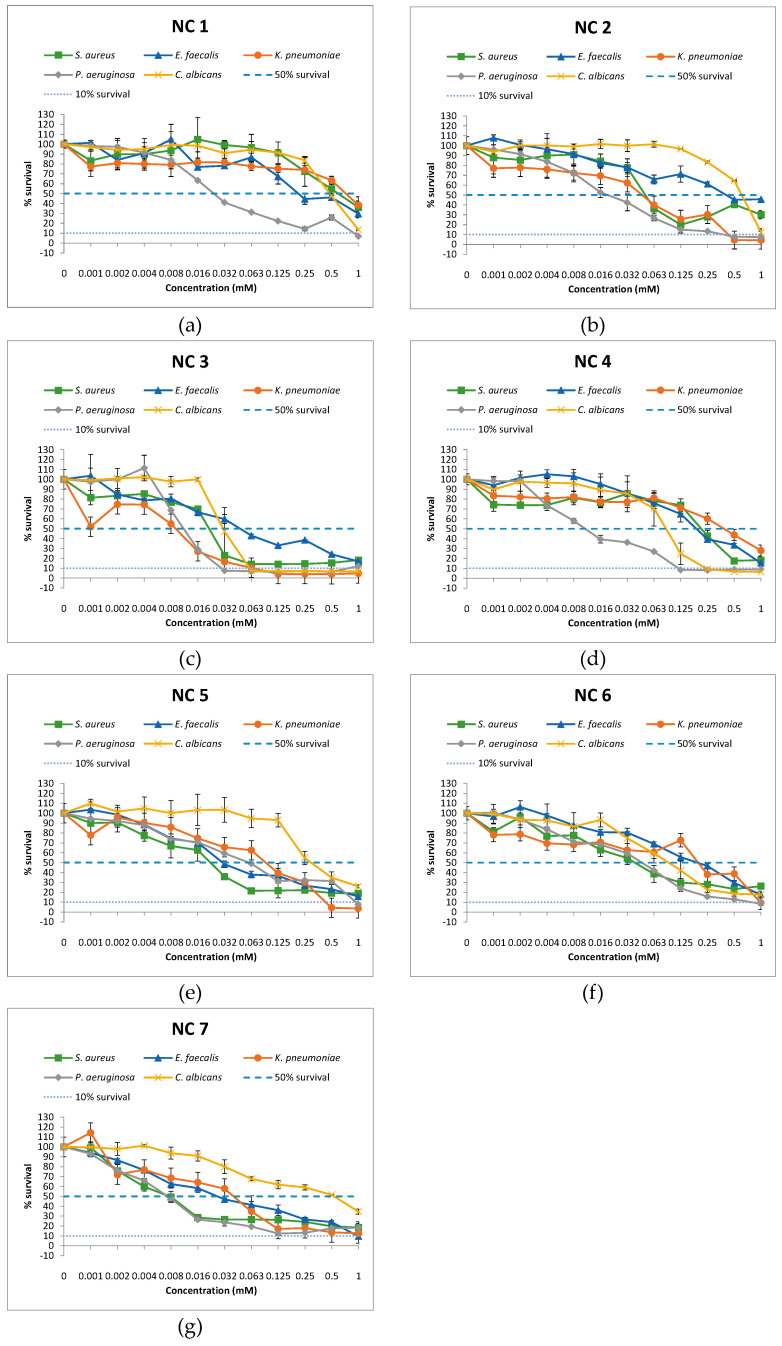
Percentage of microbial survival in the presence of nicotinamide compounds (NC) in the concentration range 0.001–1 mM: (**a**) NC **1**; (**b**) NC **2**; (**c**) NC **3**; (**d**) NC **4**; (**e**) NC **5**; (**f**) NC **6**; (**g**) NC **7**.

The results of our study indicated that examined compounds (NC **1**–**7**) exhibited a wide spectrum of growth inhibition against tested reference bacteria and yeast, depending on concentrations. The MIC_50_ values of the tested compounds for gram-positive bacterial strains ranged from 0.008 to 1 mM, while none of the seven tested compounds achieved MIC_90_ for *S. aureus* and *E. faecalis* (Figure 1 and Table 2). Similar to gram-positive bacteria, the MIC_50_ for *P. aeruginosa* and *K. pneumoniae* ranged from 0.008 to 1 mM. However, MIC_90_ for *P. aeruginosa* achieved by NC **1**–**6** was 1, 0.5, 0.032, 0.125, 1, and 1 mM, respectively. NC **2**, NC **3**, NC **5**, and NC **6** showed MIC_90_ for *K. pneumoniae* at values of 0.5, 0.063, 0.5, and 1 mM, respectively. Treatment with NC **7** did not result in 90% growth inhibition for any bacterial strain. A 50% growth reduction for *C. albicans* treated with NC **1**–**7** was achieved at concentrations of 1, 1, 0.063, 0.125, 0.5, 0.125, and 1 mM, respectively, while 90% growth inhibition was achieved with NC **3** and NC **4** at concentrations 0.063 and 0.25 mM, respectively. Due to the undefined MIC_90_ values for all compounds whose efficacy was evaluated within the concentration range of 0.001–1 mM, further analyses for the mentioned category were not conducted.

**Figure 2:**
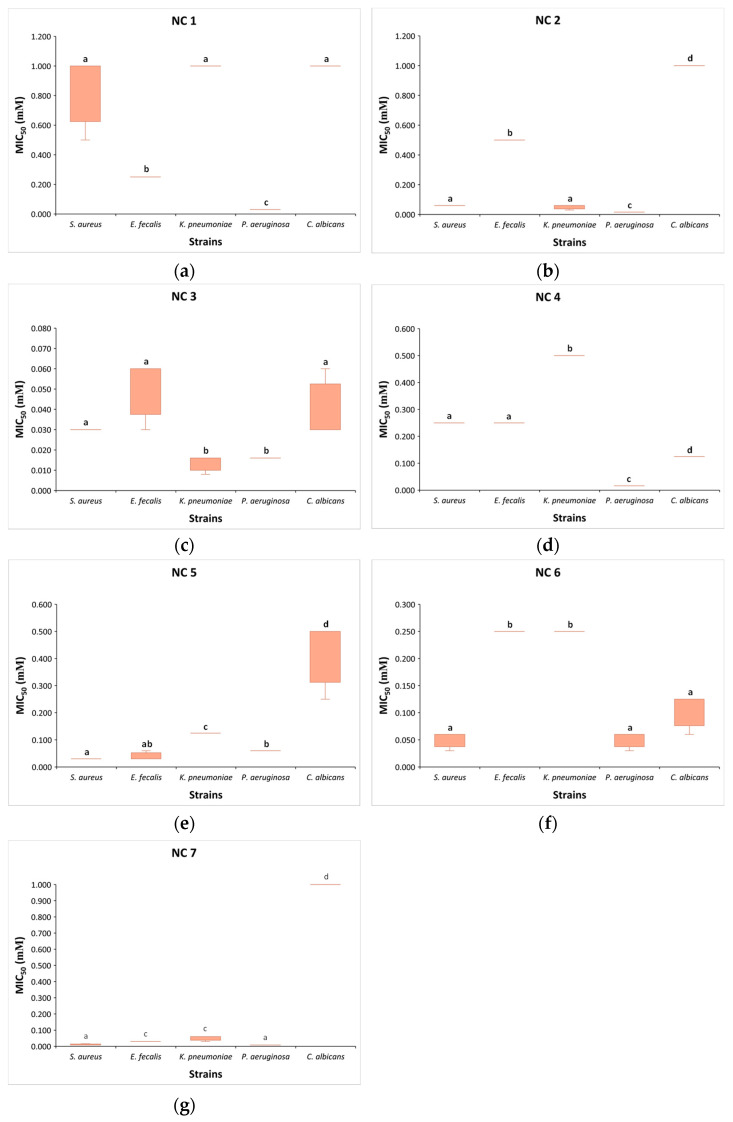
Box plots of nicotinamide compounds (NC) concentrations (mM) leading to 50% growth inhibition (MIC_50_) in *S. aureus*, *E. faecalis*, *K. pneumoniae*, *P. aeruginosa*, and *C. albicans*: (**a**) NC **1**; (**b**) NC **2**; (**c**) NC **3**; (**d**) NC **4**; (**e**) NC **5**; (f) NC **6**; (**g**) NC **7**. Kruskal–Wallis test with post hoc pairwise comparison and applied Bonferroni correction was used to compare MIC_50_ values among tested microorganisms. The same latter above box plot indicates no differences between groups (*p* > 0.05).

Based on the previously stated results, we can observe that different concentrations of the same compound lead to a 50% reduction in the growth of the tested microorganisms. *P. aeruginosa* was the most susceptible to most compounds, while *C. albicans* was the least susceptible. In comparison to *E. faecalis*, NC **1** at an eightfold lower concentration (0.032 mM) leads to a 50% growth reduction in *P. aeruginosa*, as well as at 32 times lower concentration compared to *S. aureus*, *K. pneumoniae*, and *C. albicans* (*p* < 0.05) (Figure 2). Similarly, NC **2** at a four times lower concentration (0.016 mM) effectively induces a 50% growth reduction in *P. aeruginosa* compared to *S. aureus* and *K. pneumoniae*, at a concentration 32 times lower compared to *E. faecalis* and 64 times lower compared to *C. albicans* (*p* < 0.05). NC **3** equally inhibited the growth of *P. aeruginosa* and *K. pneumoniae* at a concentration of 0.016 mM, compared to *S. aureus*, *E. faecalis*, and *C. albicans*, where double or quadruple concentrations were required for the 50% growth inhibition of these microorganisms (*p* < 0.05). Although NC **4** demonstrated the most pronounced effect on *P. aeruginosa*, it is noteworthy that this compound exhibits more potent activity against *C. albicans* compared to *S. aureus* and *E. faecalis*. Additionally, for *K. pneumoniae*, double (0.25 mM) and quadruple concentrations (0.5 mM) were required for 50% growth inhibition, respectively (*p* < 0.05). Unlike the previously mentioned compounds, NC **5** exhibited the most pronounced effect on gram-positive bacteria, where a concentration of 0.03 mM effectively reduced the growth of *S. aureus* and *E. faecalis*. It is interesting that NC **6**, at a concentration of 0.06 mM, equally effectively inhibited the growth of *S. aureus*, *P. aeruginosa*, and *C. albicans*. For a 50% growth inhibition of *K. pneumoniae* and *E. faecalis*, a quadruple concentration of NC **6**, compared to the three aforementioned strains, was required. NC **7**, compared to other nicotinamide compounds, effectively led to 50% growth inhibition in *P. aeruginosa* and *S. aureus* at a markedly low concentration of 0.008 mM. At a concentration of 0.016 mM, the mentioned compound achieved the same effect in *E. faecalis* and *K. pneumoniae*, whereas *C. albicans* required a concentration of 1 mM for 50% growth inhibition.

In comparison to other similar studies that investigated nicotinamide derivates, our research showed similar or even more promising results. Kafa et al. demonstrated that 2-chloro-N-(2-chlorophenyl)nicotinamide, 2-chloro-N-(3-chlorophenyl)nicotinamide, and 2-chloro-N-(4-chlorophenyl)nicotinamide exhibit moderate antibacterial activity (37.4–74.8 µM) against reference strains of *Staphylococcus aureus*, *E. faecalis, P. aeruginosa*, and *K. pneumoniae*. Notably, N-(2-bromophenyl)-2-chloronicotinamide was highly effective against *E. faecalis* with a MIC of 32 µM [55]. In our study, compounds NC **5** and NC **7** achieved the same MIC_50_ against *E. faecalis*, with NC **7** being more potent against *S. aureus*, showing an MIC_50_ of 0.008 mM. Our compounds were particularly effective against gram-negative strains, especially *P. aeruginosa*, with four compounds showing MIC_50_ values below 0.032 mM. Dang et al. found that pyridoxinium and nicotinium dithiophosphates had strong antibacterial activity (10–20 µM) against *S. aureus* and activity at higher concentrations (MIC≥ 320 µM) against *P. aeruginosa* and *K. pneumoniae* [56]. In our research, only NC **7** had a lower MIC_50_ against *S. aureus* (0.008 mM) compared to Dang et al.s’ findings. However, aside from NC **5** and NC **6**, all other compounds exhibited lower MIC_50_ values against *P. aeruginosa* (0.008–0.032 mM), while NC **3** exhibited MIC_50_ of 0.016 mM against *K. pneumoniae*. A notable antibacterial effect of a newly synthesized derivative 1-methyl- N’-(hydroxymethyl)nicotinamide against *E. faecalis*, *S. aureus, E. coli*, and *P. aeruginosa* was determined in the study of Adamiec et al. with *E. faecalis* being the most susceptible strain (MIC = 0.51 mg/mL) [57]. In comparison to this finding, a MIC_50_ of the most effective compound to *E. faecalis* in our research, NC **3**, is even 100 times lower. Additionally, while *P. aeruginosa* was the most resistant strain in the study by Adamiec et al. (MIC = 2.05 mg/mL); all seven compounds in our research displayed significantly lower MIC_50_ values against this strain, ranging from 0.0024 to 0.0224 mg/mL.

Alongside the recent work by Wang and colleagues, which highlighted the potent fungicidal activities of several nicotinamide derivatives modified from the analog boscalid [58], numerous latest studies have also reported promising effects of nicotinamide derivatives on fungal strains. The MIC value of novel N-(2-(2-hydrazinyl-2-oxoethylamino)-2-oxoethyl)-nicotinamide synthesized by Moustafa et al. was 160 µg/mL against *C. albicans* [59]. Whereas MIC_50_ of our NC **5** was to some extent above this value (0.1752 mg/mL), compounds NC **3**, NC **4**, and NC **6** showed remarkably lower MIC_50_ values against this strain (0.0101, 0.0438, and 0.0383 mg/mL, respectively). In the study by Kafa et al., the lowest MIC for *C. albicans* (64.1 µM) was achieved by N-(2-bromophenyl)-2-chloronicotinamide [56], which is slightly above the MIC_50_ (0.063 mM) of the compound with the lowest MIC_50_ for this fungal strain in our research, NC **3**. However, Ni et al. demonstrated that 2-amino-N-(3-isopropylphenyl)nicotinamide has antifungal activity against two fluconazole-susceptible strains with MIC values ranging from 0.125 to 0.5 µg/mL [60], which is significantly lower in comparison to our lowest MIC_50_ value of compound NC **3** (0.0101 mg/mL) that exhibits the best antifungal activity among all compounds examined in our study. Finally, in the research conducted by Xing et al., the inhibitory effect of commercial nicotinamide on *C. albicans* SC5314 was assessed, yielding a MIC_50_ value of 20 mM [19]. This is considerably higher than the MIC_50_ values of our compounds NC **3**, NC **4**, NC **5**, and NC **6** (0.032 mM, 0.125 mM, 0.5 mM, and 0.125 mM, respectively). Xing et al. also showed that nicotinamide exerts similar inhibitory effects on both fluconazole-resistant and fluconazole-susceptible *C. albicans* strains and enhances fluconazole activity against fluconazole-resistant strains (20 mM and 5 mM, respectively) [19]. Considering these findings and our results, further investigation into the synergistic effects of our compounds NC **1**–**7** with fluconazole could be valuable. Moreover, Xing et al. demonstrated that the combination of nicotinamide and fluconazole has a pronounced effect against *C. glabrata* and *C. krusei* isolates, which are highly resistant to fluconazole, significantly lowering the MIC for this drug. This insight should guide our future investigations on the impact of our newly synthesized compounds NC **1**–**7** on emerging *Candida* strains.

### 3.4. Target Fishing

This study describes the antimicrobial activity of nicotinamide thiocarbohydrazones for the first time. Notably, in our prior investigation, compounds sharing structural similarity with NC **1**–**7** were identified as potent against *M. tuberculosis* [61]. A pharmacophoric similarity search using PharmMapper identified several proteins as potential antimicrobial targets for the investigated compounds. NC **3**, in particular, exhibited promising antibacterial potential against bleomycin resistance protein (BRP), HTH-type transcriptional regulator QacR, and streptogramin A acetyltransferase, as determined by *z*-score and biological relevance (Table 3). Significantly, all three proteins belong to antibiotic-binding proteins, and bacteria use them as defense mechanisms against established antibiotics. Indeed, while statistically significant differences in the concentrations of NC **3** required to inhibit the growth of the tested microorganisms have been identified, it is noteworthy that the concentration of NC **3** leading to a 50% growth inhibition (0.016–0.064 mM) is the lowest compared to the remaining six compounds. Therefore, NC **3**, which contains nicotinamide and 2-pyridil monothiocarbohydrazone moieties connected via an imino linker, can be considered as the compound with the most promising antibacterial activity in our research.

**Figure 3:**
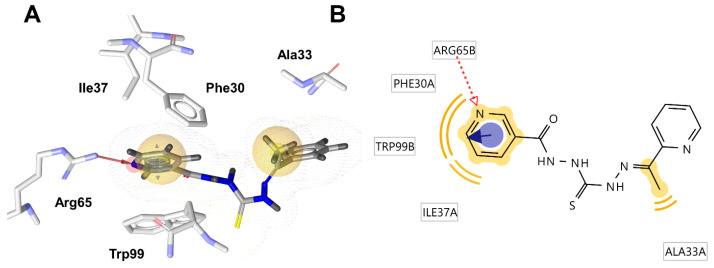
(**A**) Binding mode of NC **3** within the active site of bleomycin resistance protein (1EWJ). The ligand’s pharmacophores are denoted by yellow spheres for hydrophobic interactions, blue circles for π-π stacking, and red arrows indicating hydrogen bond acceptor (HBA) interactions with the specified amino acid residues; (**B**) Corresponding 2D ligand interaction diagram (LID).

We then conducted molecular docking calculations to explore the binding mode of nicotinamide thiocarbohydrazones within the identified antimicrobial targets. The best docking solution of NC **3** within the active site of BRP exhibited a binding affinity of −7.5 kcal/mol. In this configuration, the nicotinamide aromatic moiety engaged in hydrophobic and π-π stacking interactions with Trp99 and Phe30, resembling the interactions observed between the thiazolium rings of bleomycin and the BRP in the corresponding crystal structure. Additionally, the pyridine nitrogen established a hydrogen bond acceptor (HBA) interaction with Arg65 (Figure 3). According to CARD database analysis, the BRP-encoding gene is found in *P. aeruginosa* and *K. pneumoniae*, which may explain the higher sensitivity of these two strains to NC **3** compared with *S. aureus* and *E. faecalis* (https://card.mcmaster.ca/ontology/37586; accessed on 25th July 2024) [65].

**Figure 4:**
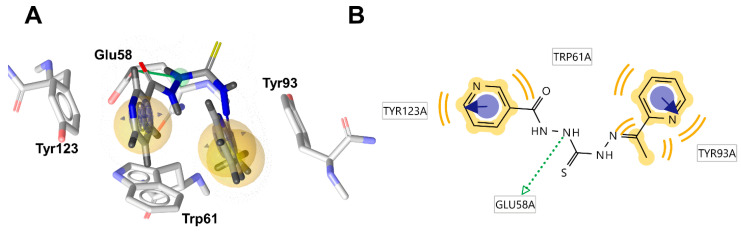
(**A**) Binding mode of NC **3** within the active site of HTH-type transcriptional regulator QacR (1JT6). The ligand’s pharmacophores are labeled with yellow spheres for hydrophobic interactions, blue circles for π-π stacking, and green arrow for hydrogen bond donor (HBD) interactions with the indicated amino acid residues; (**B**) Corresponding 2D ligand interaction diagram (LID).

Transcriptional regulator protein QacR was identified as another potential target. NC **3** demonstrated high affinity (−8.6 kcal/mol) to this protein, attributed to hydrophobic and stacking interactions of the ligand with Trp61, Tyr93 andTyr123, along with a HBD interaction with Glu58 (Figure 4).

Streptogramin A acetyltransferase (VatD) emerges as another suggested molecular target for NC **3** in bacterial cells. The ligand exhibits moderate binding affinity (−6.9 kcal/mol), attributed to several hydrophobic and hydrogen-bonding interactions with the active site residues (Figure S36).

Finally, the affinity of NC **3** to DHFR was confirmed through molecular docking simulations. The ligand engages in HBA interactions with Leu24 and Gln28, π-π stacking interactions with Phe31, and a network of hydrophobic interactions involving several amino acid residues and NADPH (Figure S37). These interactions collectively contribute to a binding affinity of −7.4 kcal/mol.

Swiss-ADME predictions indicate that NC **3** is a highly water-soluble compound with excellent gastrointestinal (GI) absorption, low blood-brain barrier (BBB) permeability, no affinity to P-glycoproteins and cytochromes, and no violations of Lipinski’s Rule of Five (RO5). Moreover, the compound does not exhibit PAINS fragments and represents a lead-like structure.

## 4. Conclusions

Our study presents an efficient strategy for synthesizing new nicotinamide derivatives with promising antibacterial and antifungal effects. The growth of gram-negative bacterial strains was most significantly inhibited by NC **3** at 0.016 mM, while NC **5** was most effective against gram-positive bacteria at 0.03 mM. NC **4** exhibited the highest potency against *C. albicans*, completely inhibiting its growth at concentrations below 1 mM. The NC **3**, which contains nicotinamide and 2-pyridil monothiocarbohydrazone moieties connected via an imino linker, leads to a 50% growth inhibition at the lowest concentration compared to the remaining six compounds, thus representing the compound with the most promising antibacterial activity in our research. The high affinity and pronounced pharmacophoric similarity of NC **3** with several proteins associated with well-known mechanisms of AMR suggest that this class of compounds has the potential to enhance or restore the activity of established antibiotics when co-administered. *P. aeruginosa* and *K. pneumoniae* are more sensitive to NC **3** compared to *S. aureus* and *E. faecalis*, likely due to the presence of BRP-encoding genes. We have created a library of related compounds for further exploration, aiming to improve the antimicrobial properties of nicotinamide-based compounds and investigate their antimicrobial activities against clinical strains of emerging drug-resistant pathogens and biofilm properties, as well as their antioxidant and cytotoxic effects.

## Figures and Tables

**Table 1:** Name and structure of synthesized nicotinamides NC **1**–**7** (refer to Scheme 2 or Scheme 3).

	Compounds	Ar	R
NC **1**	1-[(2-hydroxyphenyl)methyl]-5-(pyridine-3-carbonyl)dihydrazide thiocarbonyl acid	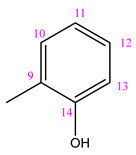	H
NC **2**	1-(2-pyridinylmethylene)-5-(pyridine-3-carbonyl)dyhidrazide thiocarbonyl acid	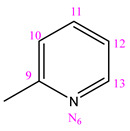	H
NC **3**	1-[1-(2-pyridinyl)ethylidene]-5-(pyridine-3-carbonyl)dyhidrazide thiocarbonyl acid	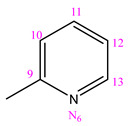	CH_3_
NC **4**	1-(8-hinolylmethylene)-5-(pyridine-3-carbonyl)dyhidrazide thiocarbonyl acid	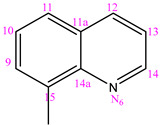	H
NC **5**	1-(2-hynolilmethylene)-5-(pyridine-3-carbonyl)dyhidrazide thiocarbonyl acid	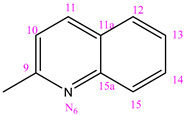	H
NC **6**	1-[(8-hydroxy)-2-hynolilmethilen]-5-(pyridine-3-carbonyl)dyhidrazide thiocarbonyl acid	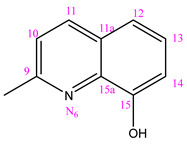	H
NC **7**	1-(phenyl-methylen)-5-(pyridine-3-carbonyl)dyhidrazide thiocarbonyl acid	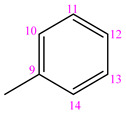	H

Full characterization of NC **1**–**7** was performed using elemental analysis, FTIR, NMR, and MS instrumental techniques.

**Table 2:** MIC values of nicotinamide compounds (NC **1**–**7**) against bacterial and fungal strains evaluated by the broth microdilution method.

Compound	MIC	Microorganism									
		*S. aureus* (ATCC 6538)		*E. faecalis* (ATCC 29212)		*K. pneumoniae* (ATCC 27853)		*P. aeruginosa* (NCIMB 9111)		*C. albicans* (ATCC 24433)	
		mg/mL	mM	mg/mL	mM	mg/mL	mM	mg/mL	mM	mg/mL	mM
NC **1**	MIC_R_	>0.3154	>1	>0.3154	>1	>0.3154	>1	0.3154	1	>0.3154	>1
	MIC_50_	0.3154	1	0.0788	0.25	0.3154	1	0.0101	0.032	0.3154	1
	MIC_90_	>0.3154	>1	>0.3154	>1	>0.3154	>1	0.3154	1	>0.3154	>1
NC **2**	MIC_R_	>0.3033	>1	>0.3033	>1	0.1517	0.5	0.3033	1	>0.3033	>1
	MIC_50_	0.0194	0.064	0.1517	0.5	0.0194	0.064	0.0194	0.016	0.3033	1
	MIC_90_	>0.3033	>1	>0.3033	>1	0.1517	0.5	0.1517	0.5	>0.3033	>1
NC **3**	MIC_R_	>0.3144	>1	>0.3144	>1	0.0101	0.032	0.0101	0.032	0.3144	1
	MIC_50_	0.0101	0.032	0.0201	0.064	0.0050	0.016	0.0050	0.016	0.0101	0.032
	MIC_90_	>0.3144	>1	>0.3144	>1	0.0201	0.064	0.0101	0.032	0.0201	0.064
NC **4**	MIC_R_	>0.3504	>1	>0.3504	>1	>0.3504	>1	0.3504	1	0.1752	0.5
	MIC_50_	0.0876	0.25	0.0876	0.25	0,1752	0.5	0.0056	0.016	0.0438	0.125
	MIC_90_	>0.3504	>1	>0.3504	>1	>0.3504	>1	0.0438	0.125	0.0876	0.25
NC **5**	MIC_R_	>0.3504	>1	>0.3504	>1	>0.3504	>1	0.3504	1	>0.3504	>1
	MIC_50_	0,0112	0.032	0.0112	0.032	0.0438	0.125	0.0224	0.064	0.1752	0.5
	MIC_90_	>0.3504	>1	>0.3504	>1	0.1752	0.5	0.3504	1	>0.3504	>1
NC **6**	MIC_R_	>0.3064	>1	>0.3064	>1	0.3064	1	>0.3064	>1	>0.3064	>1
	MIC_50_	0.0196	0.064	0.0766	0.25	0.0766	0.25	0.0196	0.064	0.0383	0.125
	MIC_90_	>0.3064	>1	>0.3064	>1	>0.3064	>1	0.3064	1	>0.3064	>1
NC **7**	MIC_R_	>0.2994	>1	>0.2994	>1	0.1497	0.5	>0.2994	>1	>0.2994	>1
	MIC_50_	0.0024	0.008	0.0096	0.032	0.0147	0.06	0.0024	0.008	0.2994	1
	MIC_90_	>0.2994	>1	>0.2994	>1	>0.2994	>1	>0.2994	>1	>0.2994	>1
Levofloxacin	MIC_R_	μg/mL		μg/mL		μg/mL		μg/mL		N/A	
		0.001		4		0.125		0.5			
Amikacin	MIC_R_	μg/mL		N/A		μg/mL		μg/mL		N/A	
		16				8		16			
Fluconazole	MIC_R_	N/A		N/A		N/A		N/A		μg/mL	
										2	

NC = nicotinamide compound; MIC_R_ = minimum inhibitory concentration defined by resazurin method; MIC_50_ = minimum concentration leading to 50% growth inhibition defined by optical density measurement; MIC_90_ = minimum concentration leading to 90% growth inhibition defined by optical density measurement; N/A = not applicable.

**Table 3:** The plausible antimicrobial targets for NC **3** were identified via a pharmacophoric similarity search.

Target	PDB Code	Biological Role	*z*-Score	Reference
Bleomycin resistance protein	1EWJ	Confers defense against bleomycin-induced DNA cleavage in cells	4.10	[62]
HTH-type transcriptional regulator QacR	1JT6	Regulates the expression of the QacA multidrug efflux pump in *S. aureus*	2.81	[63]
Streptogramin A acetyltransferase	1KHR	Xenobiotic acetyltransferase responsible for streptogramin resistance phenotype	2.12	[64]

## Data Availability

The original contributions presented in the study are included in the article/Supplementary Materials, further inquiries can be directed to the corresponding authors.

## Supplementary Materials

The following supporting information can be downloaded at: https://www.mdpi.com/article/10.3390/pharmaceutics16081084/s1, Figure S1: FTIR spectrum of NC **1**; Figure S2: ^1^H NMR spectrum of NC **1**; Figure S3: ^13^C spectrum of NC **1**; Figure S4: HSQC spectrum of NC **1**; Figure S5: MS spectrum of NC **1**; Figure S6: FTIR spectrum of NC **2**; Figure S7: ^1^H NMR spectrum of NC **2**; Figure S8: ^13^C NMR spectrum of NC **2**; Figure S9: HSQC spectrum of NC **2**; Figure S10: MS spectrum of NC **2**; Figure S11: FTIR spectrum of NC **3**; Figure S12: ^1^H NMR spectrum of NC **3**; Figure S13: ^13^C NMR spectrum of NC **3**; Figure S14: HSQC spectrum of NC **3**; Figure S15: MS spectrum of NC **3**; Figure S16: FTIR spectrum of NC **4**; Figure S17: ^1^H NMR spectrum of NC **4**; Figure S18: ^13^C NMR spectrum of NC **4**; Figure S19: HSQC spectrum of NC **4**; Figure S20: MS spectrum of NC **4**; Figure S21: FTIR spectrum of NC **5**; Figure S22: ^1^H NMR spectrum of NC **5**; Figure S23: ^13^C NMR spectrum of NC **5**; Figure S24: HSQC spectrum of NC **5**; Figure S25: MS spectrum of NC **5**; Figure S26: FTIR spectrum of NC **6**; Figure S27: ^1^H NMR spectrum of NC **6**; Figure S28: ^13^C NMR spectrum of NC **6**; Figure S29: HSQC spectrum of NC **6**; Figure S30: MS spectrum of NC **6**; Figure S31: FTIR spectrum of NC **7**; Figure S32: ^1^H spectrum of NC **7**; Figure S33: ^13^C spectrum of NC **7**; Figure S34: HSQC spectrum of NC **7**; Figure S35: MS spectrum of NC **7**; Figure S36: Binding mode of NC **3** within the active site of Streptogramin A acetyltransferase (VatD)(1KHR); Figure S37: Binding mode of NC **3** into the active site of DHFR (1DG5).
