# Supplementary material for: Synthesis and Antimicrobial Activity of Newly Synthesized Nicotinamides
Source: Pharmaceutics. 2024 Aug 18;16(8):1084. doi: 10.3390/pharmaceutics16081084 (PMC11359232; doi:10.3390/pharmaceutics16081084)
Supplement: Supplementary file 1 [file pharmaceutics-16-01084-s001.zip › pharmaceutics-3109459-supplementary.pdf]

pharmaceutics

MDPI

Supplementary Materials

Synthesis and Antimicrobial Activity of Newly Synthesized Nicotinamides

Bojana Anić Marković [1], Aleksandar Marinković [1], Jelena Antić Stanković [2,*], Stefan Mijatović [3], Ilija Cvijetić [4], Milena Simić [2] and Irena Arandjelović [3,*]

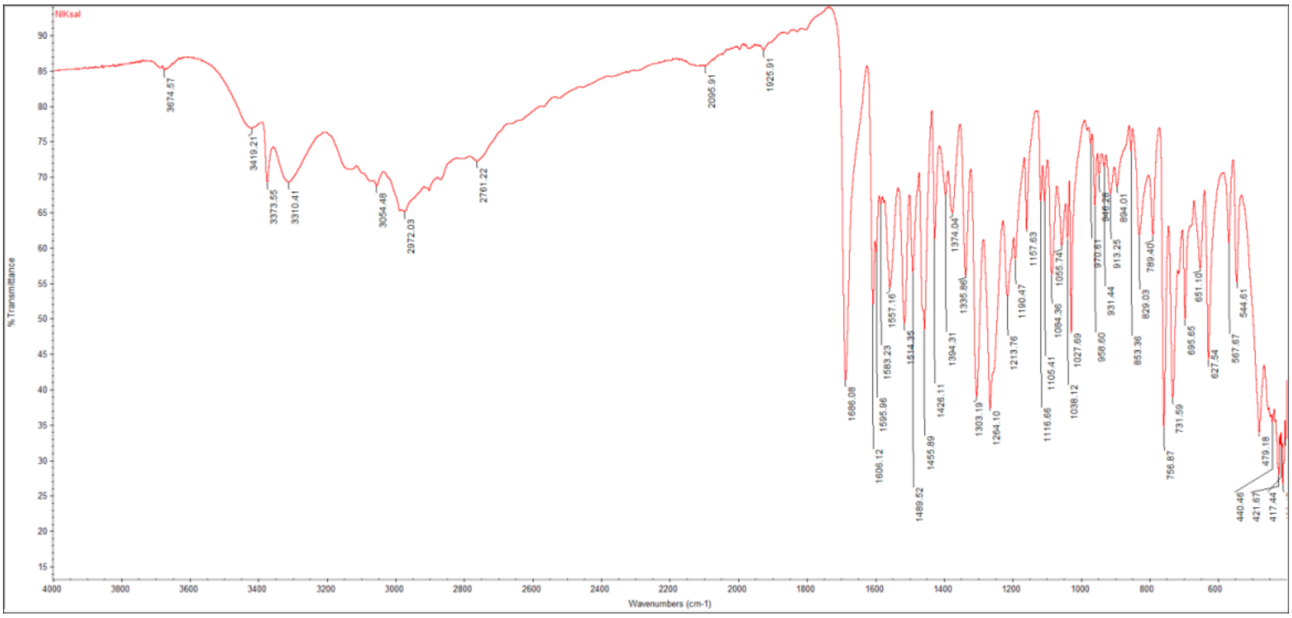

Figure S1. FTIR spectrum of NC 1.

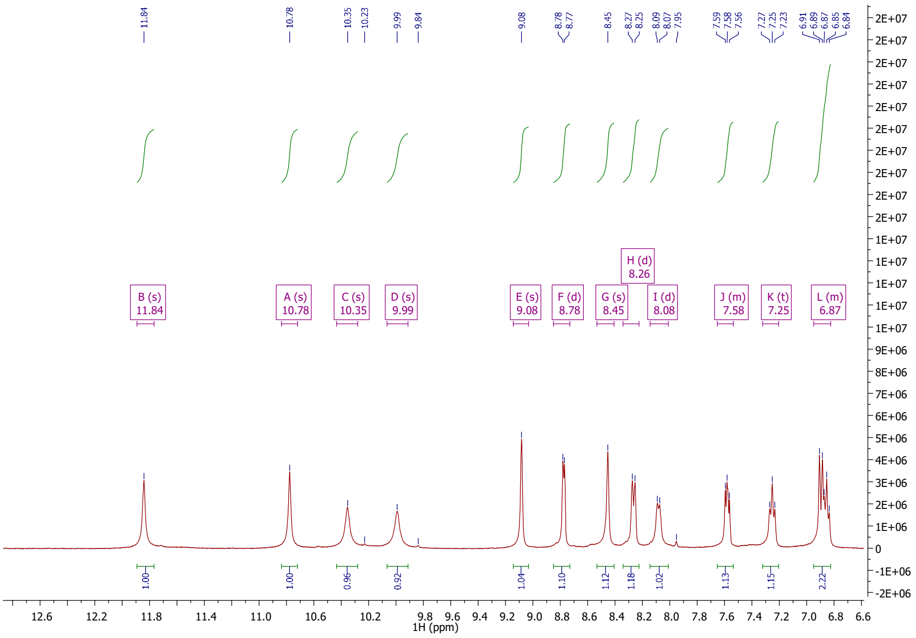

Figure S2. ^{1}H spectrum of NC 1.

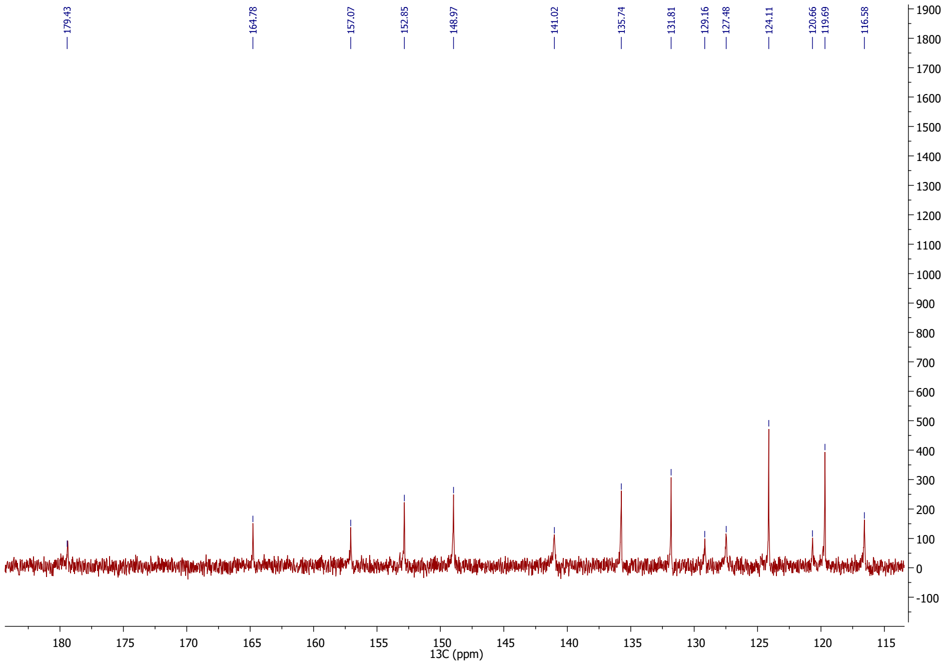

Figure S3. ^{13}C spectrum of NC 1.

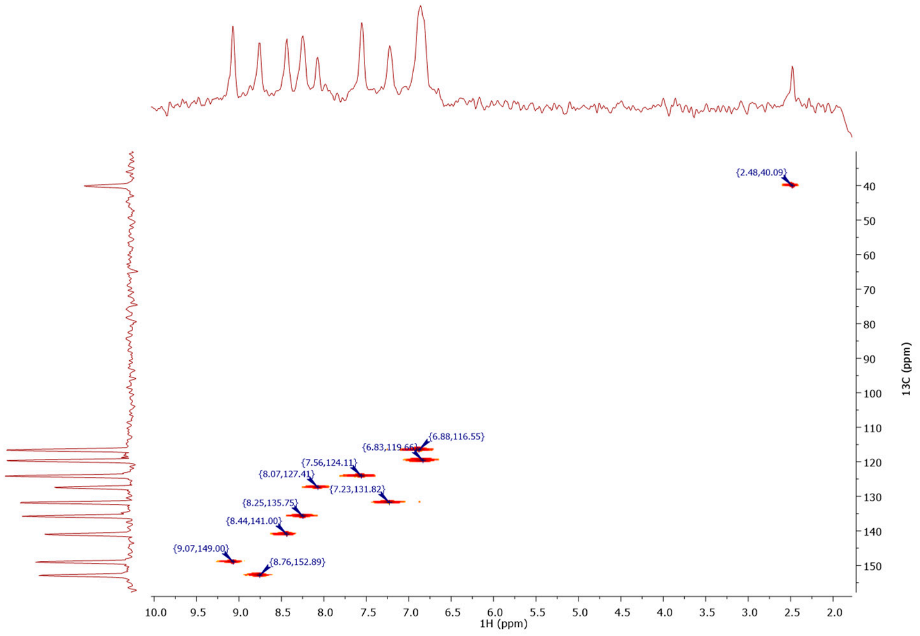

Figure S4. HSQC spectrum of NC 1.

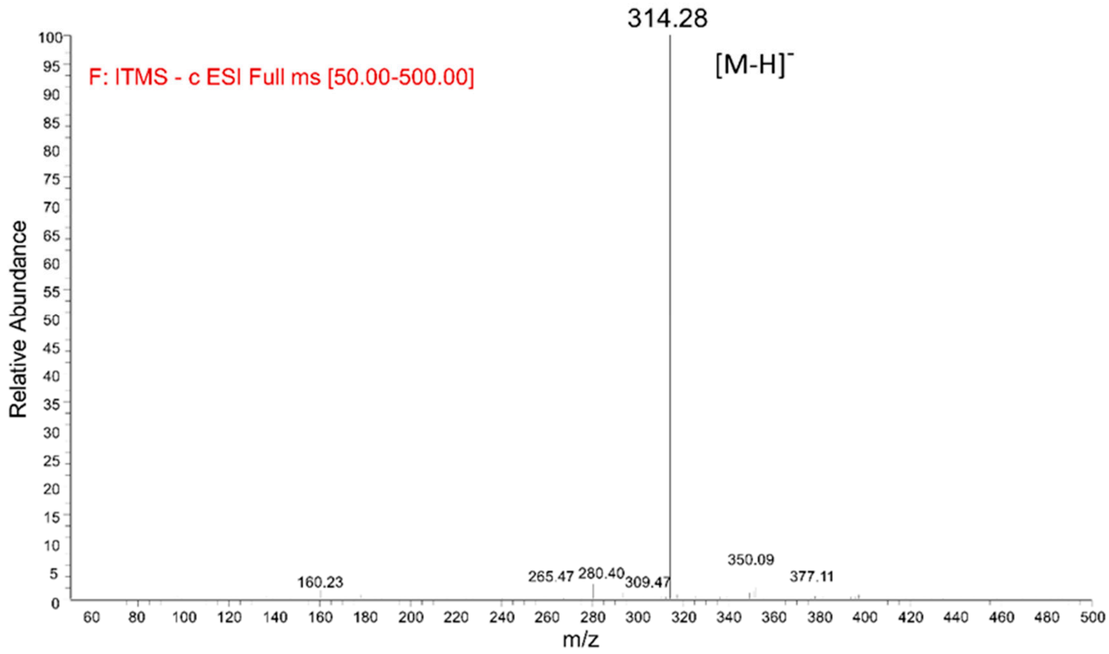

Figure S5. MS spectrum of NC 1.

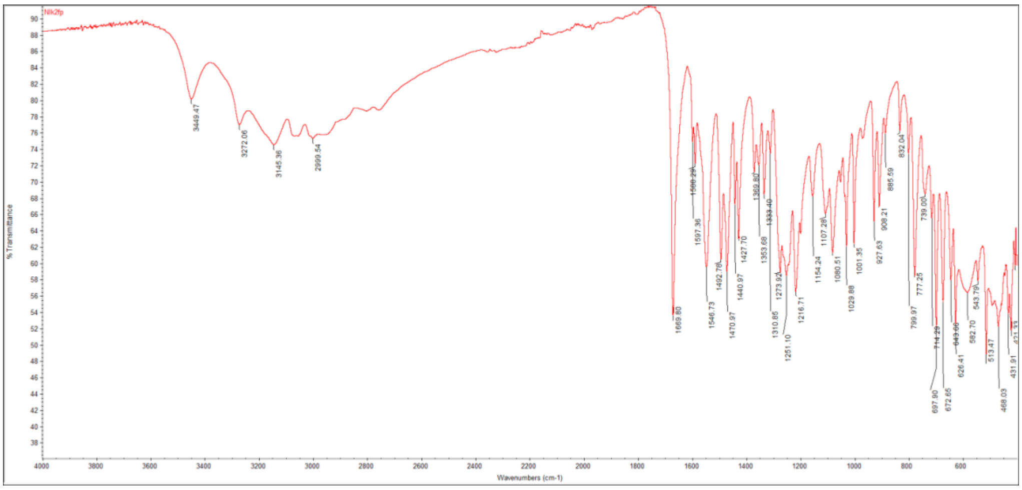

Figure S6. FTIR spectrum of NC 2.

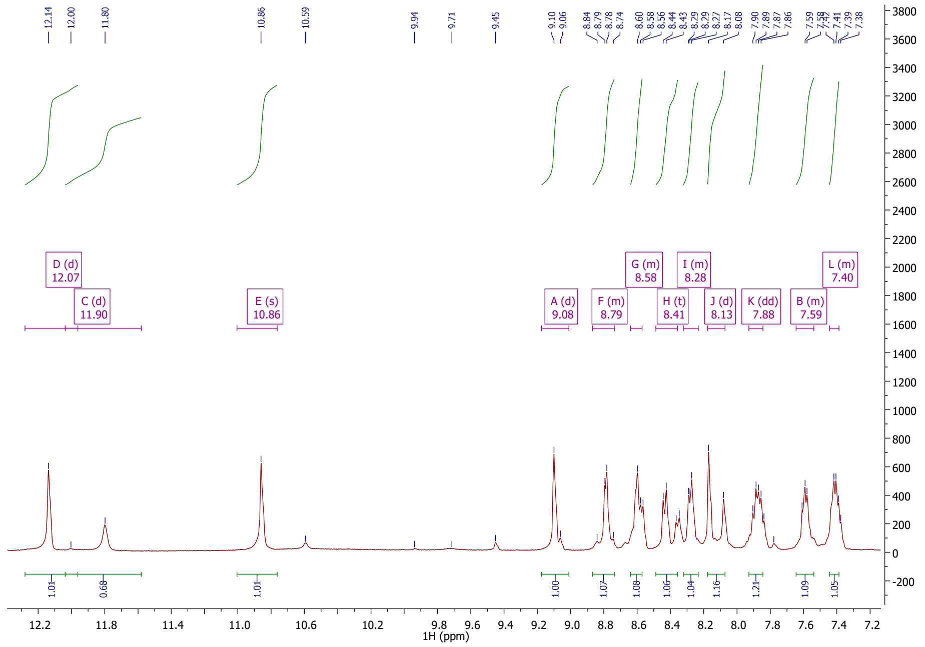

Figure S7. ^{1}H spectrum of NC 2.

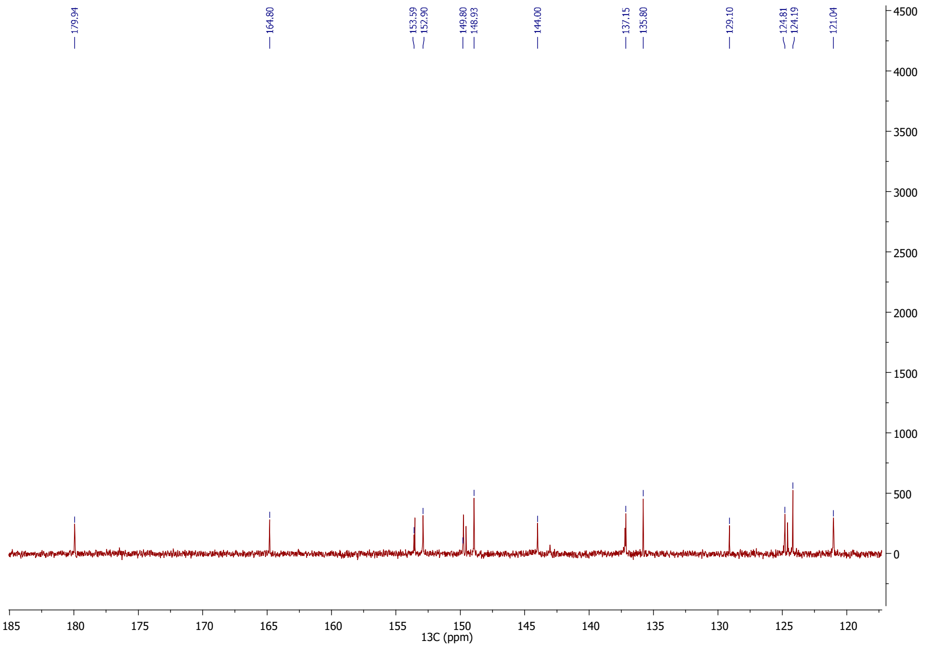

Figure S8. ^{13}C spectrum of NC 2.

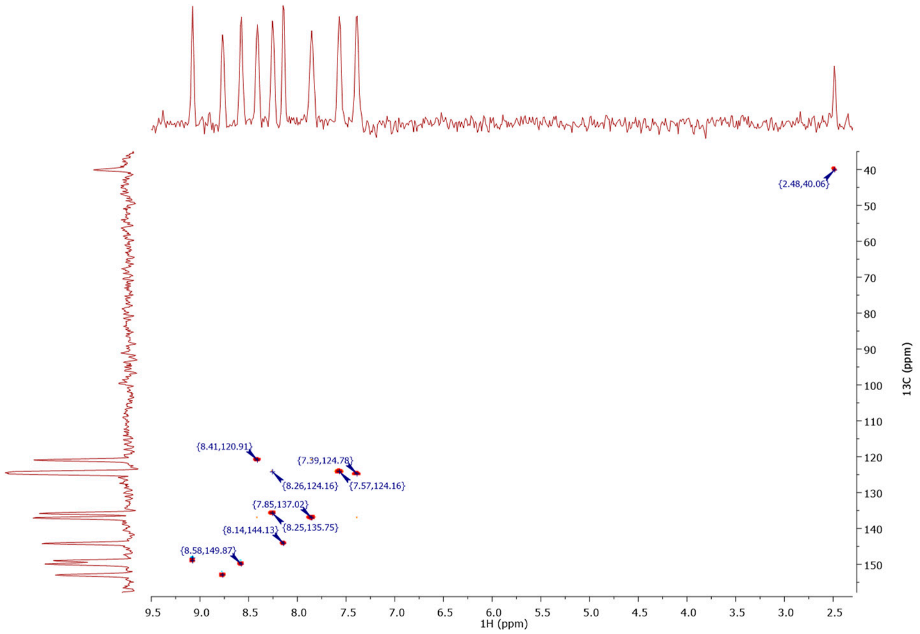

Figure S9. HSQC spectrum of NC 2**.**

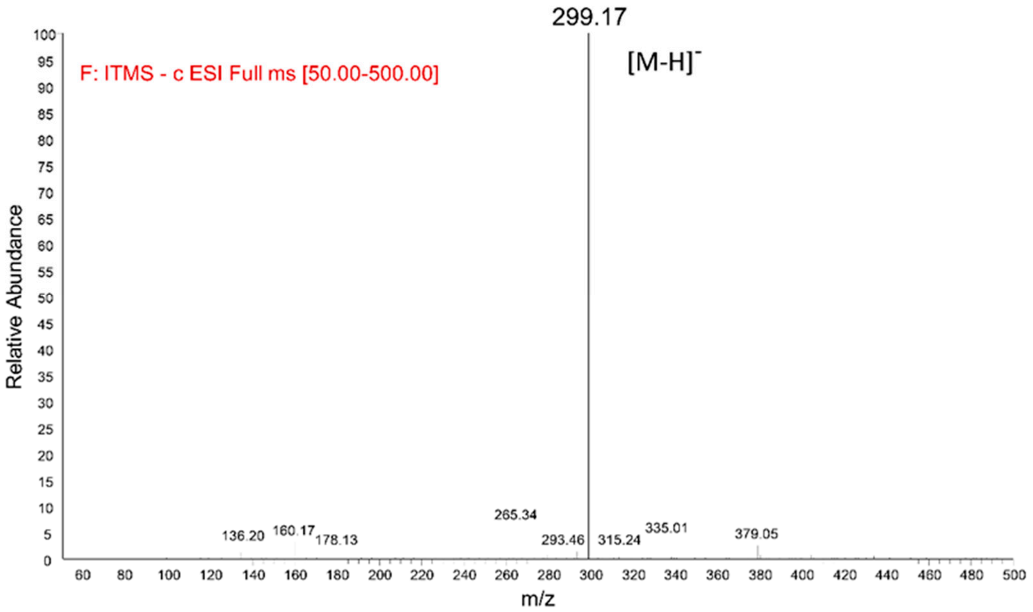

Figure S10. MS spectrum of NC 2.

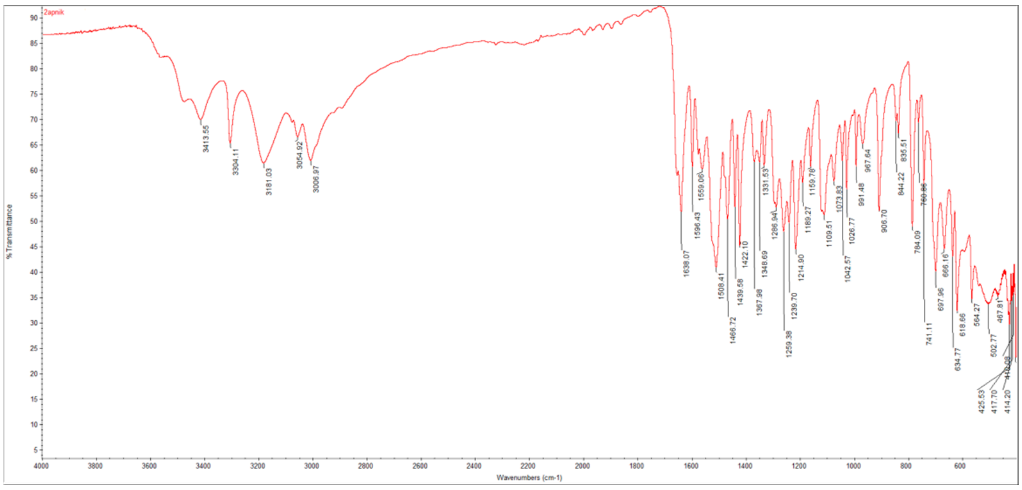

Figure S11. FTIR spectrum of NC 3.

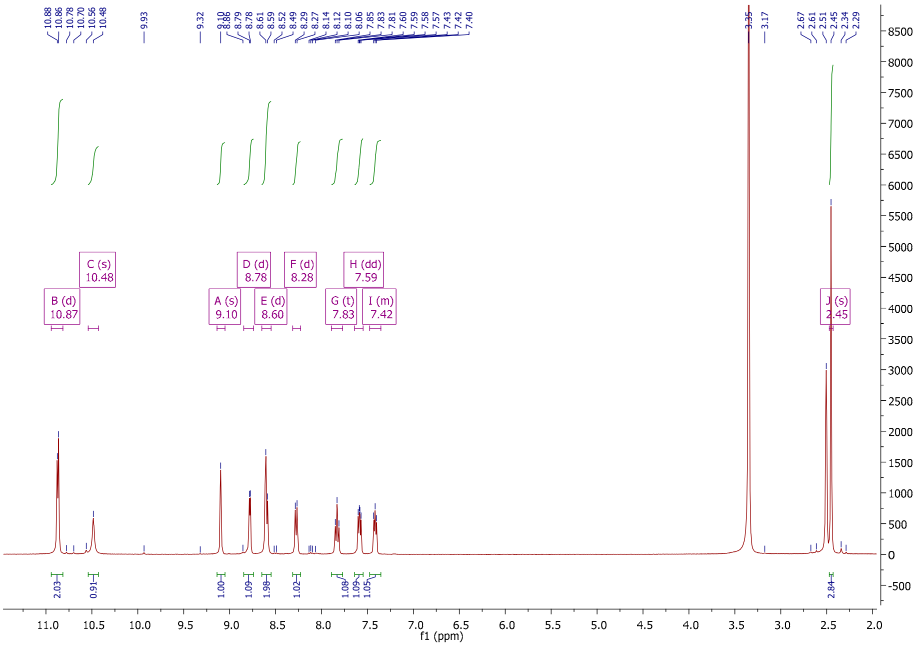

Figure S12. ^{1}H spectrum of NC 3**.**

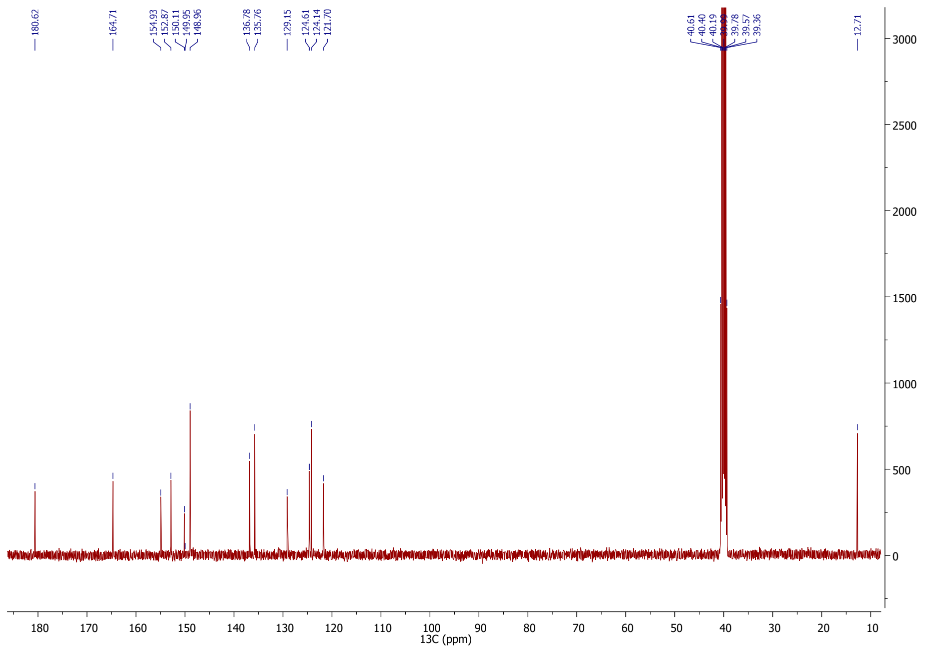

Figure S13. ^{13}C spectrum of NC 3.

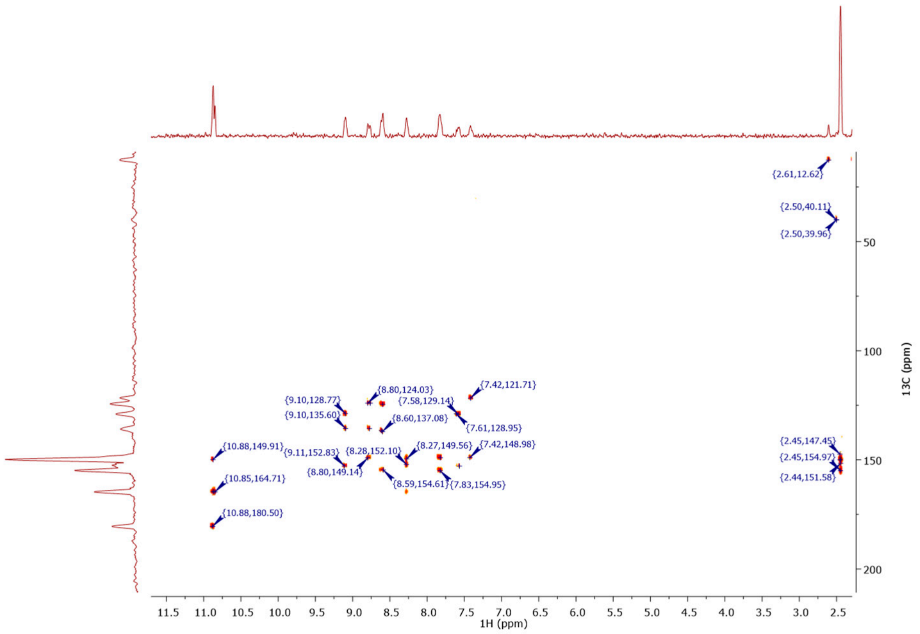

Figure S14. HSQC spectrum of NC 3.

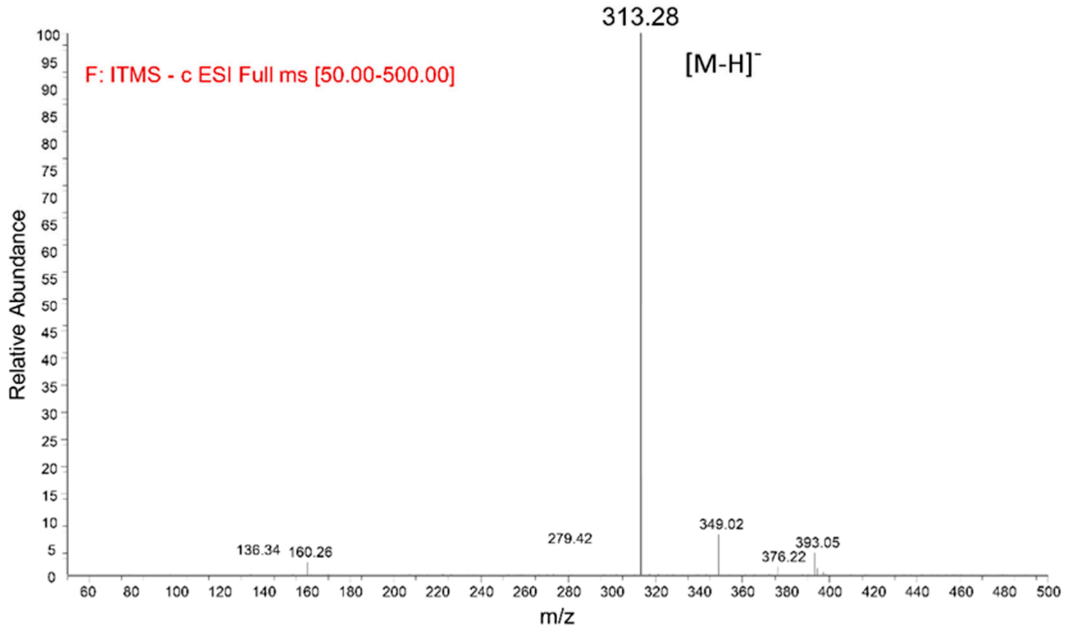

Figure S15. MS spectrum of NC 3.

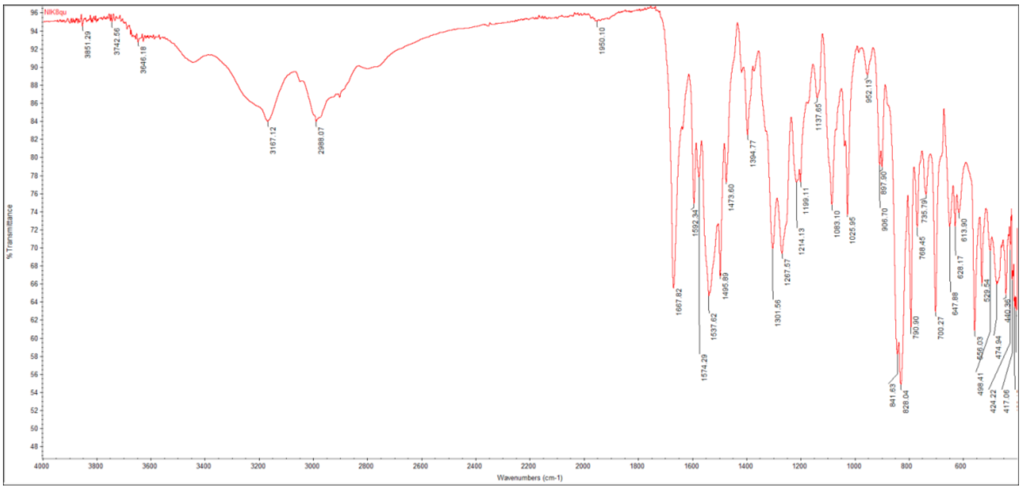

Figure S16. FTIR spectrum of NC 4.

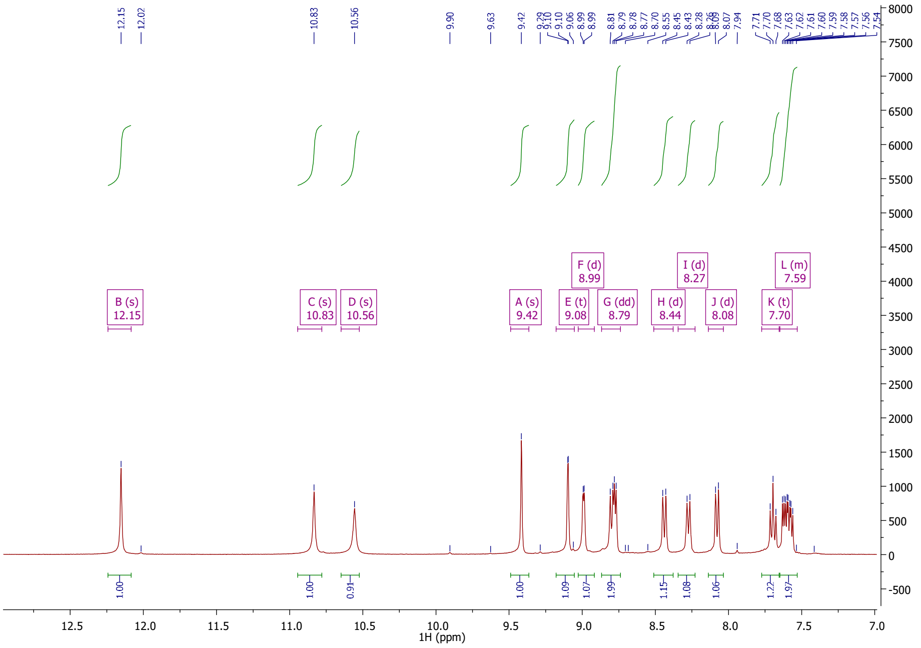

Figure S17. ^{1}H spectrum of NC 4.

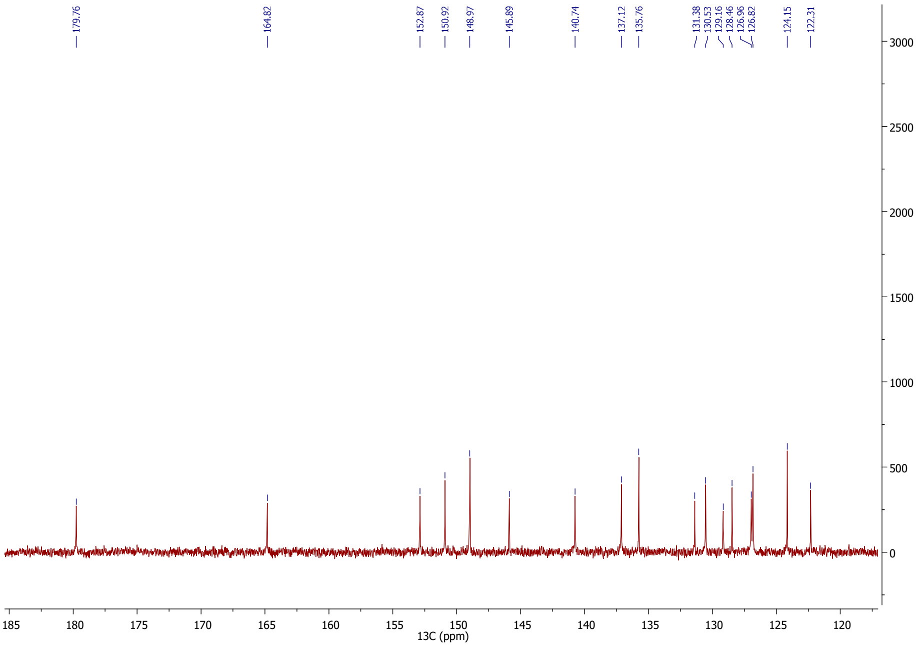

Figure S18. ^{13}C spectrum of NC 4.

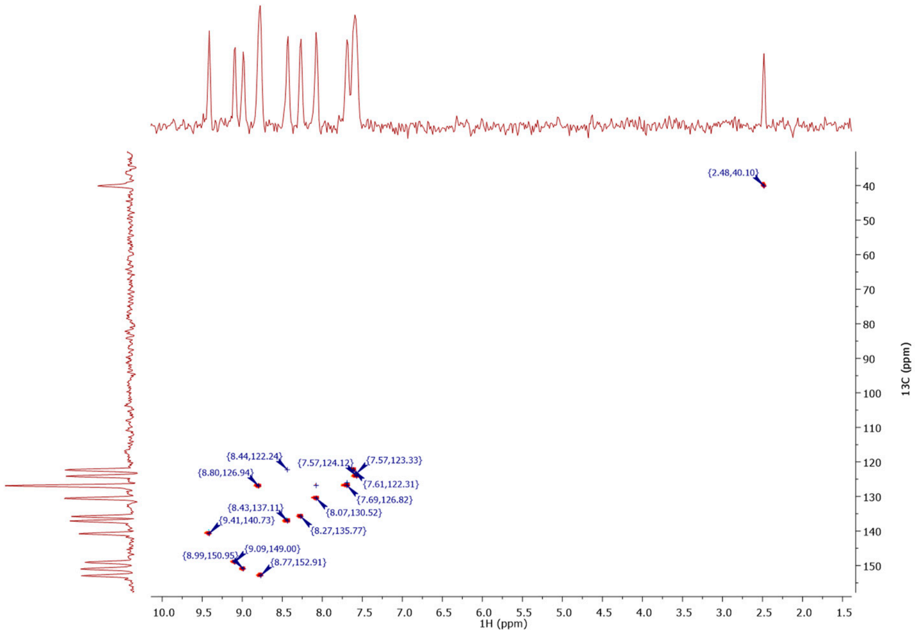

Figure S19. HSQC spectrum of NC 4.

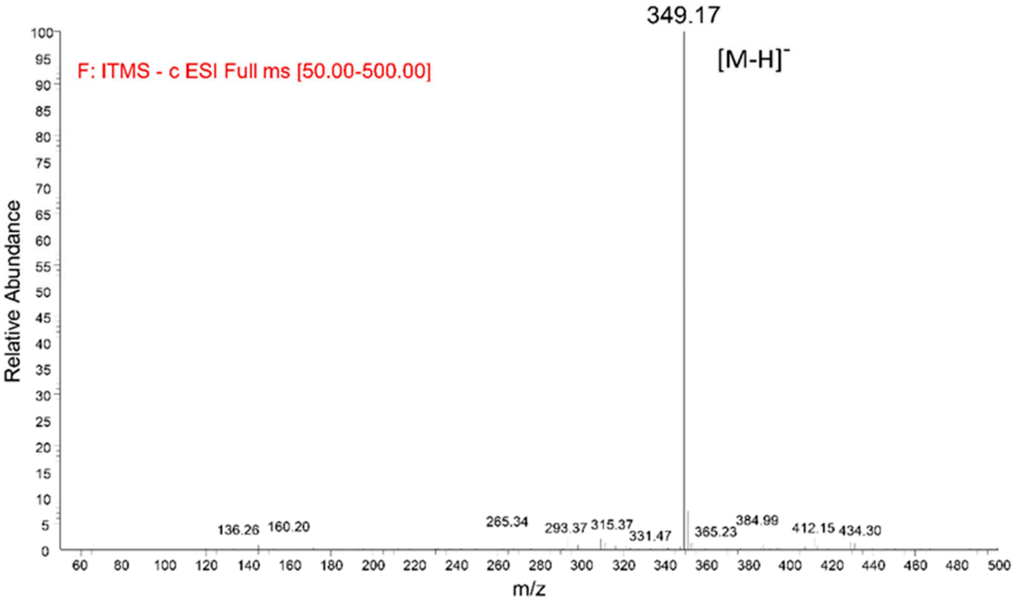

Figure S20. MS spectrum of NC 4.

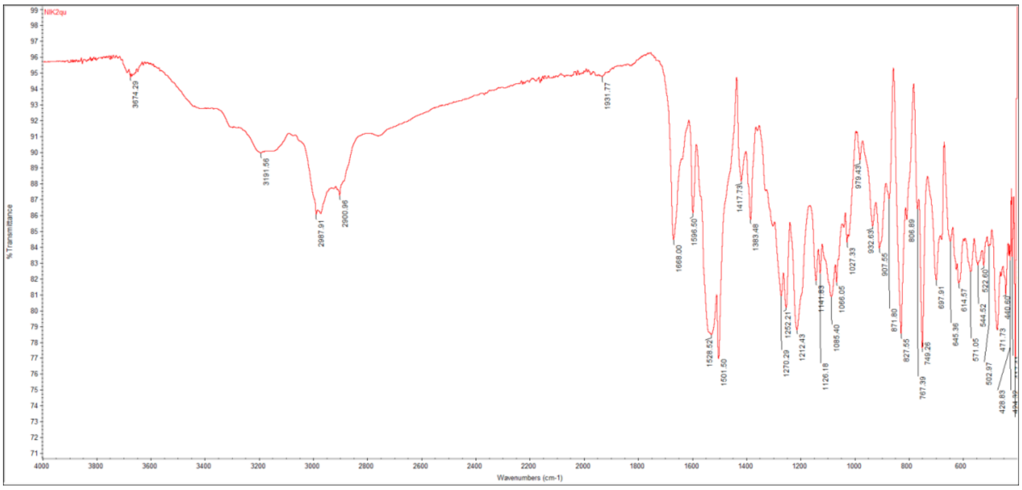

Figure S21. FTIR spectrum of NC 5.

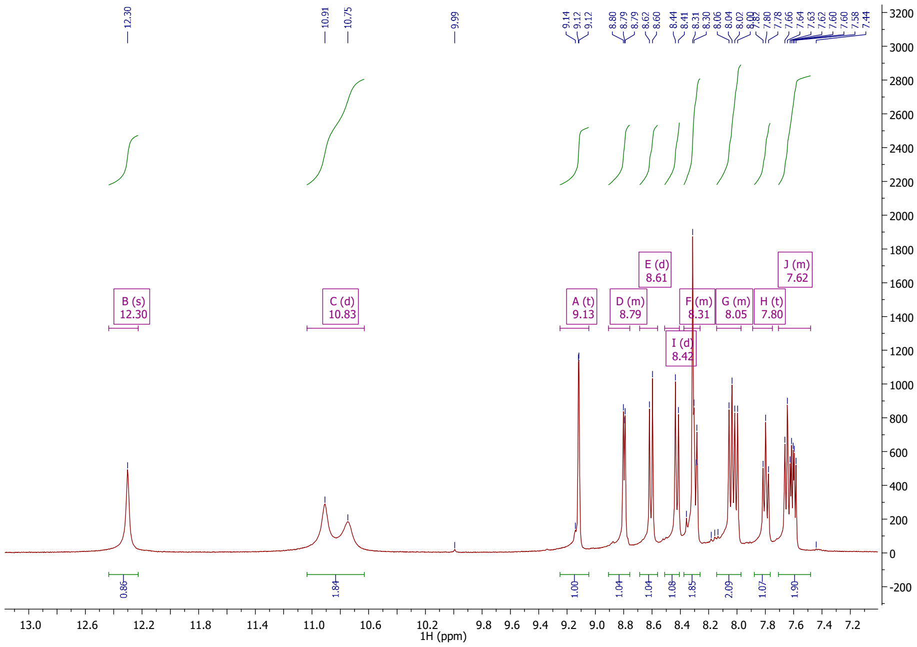

Figure S22. ^{1}H spectrum of NC 5.

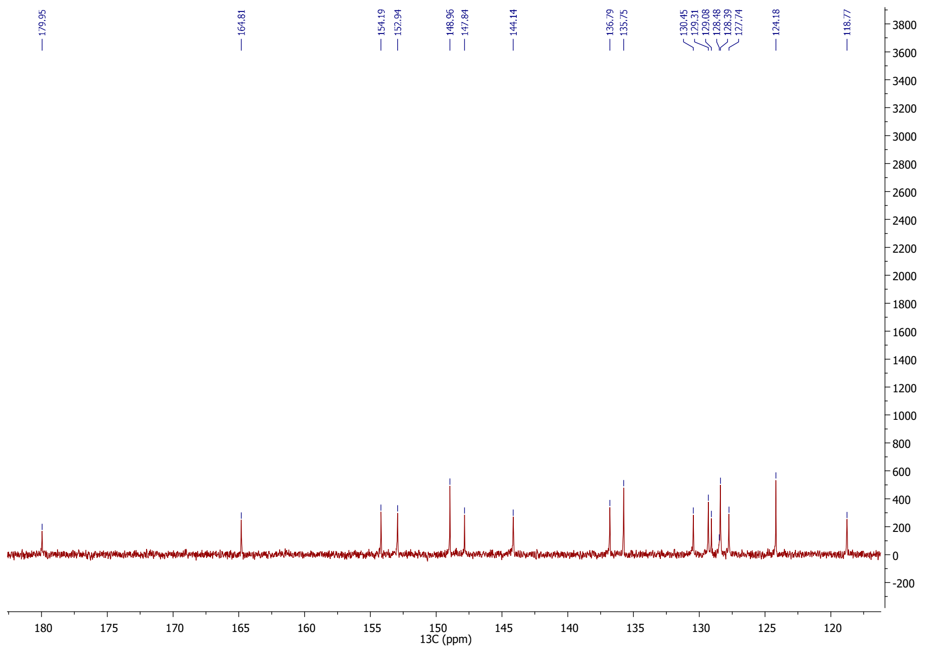

Figure S23. ^{13}C spectrum of NC 5.

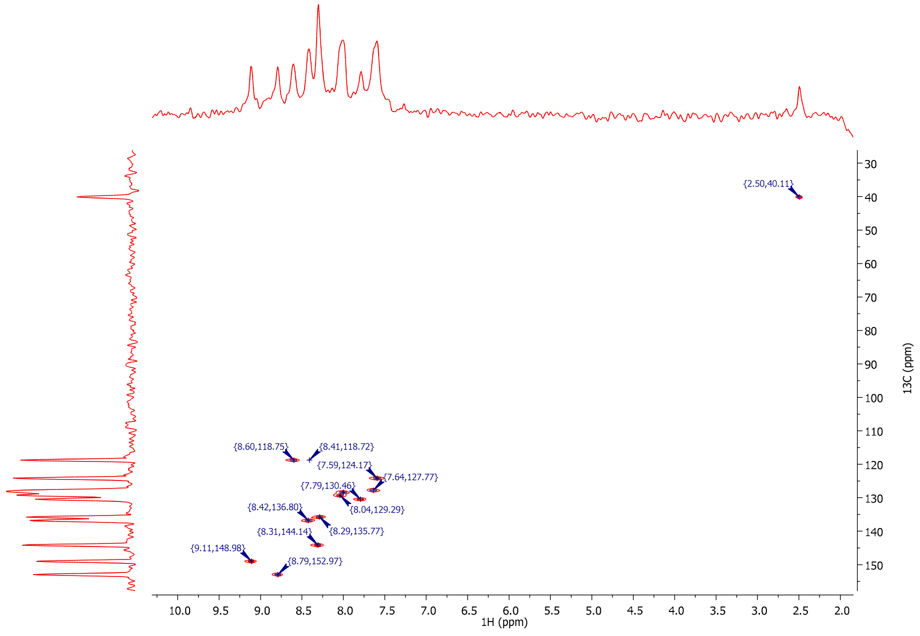

Figure S24. HSQC spectrum of NC 5.

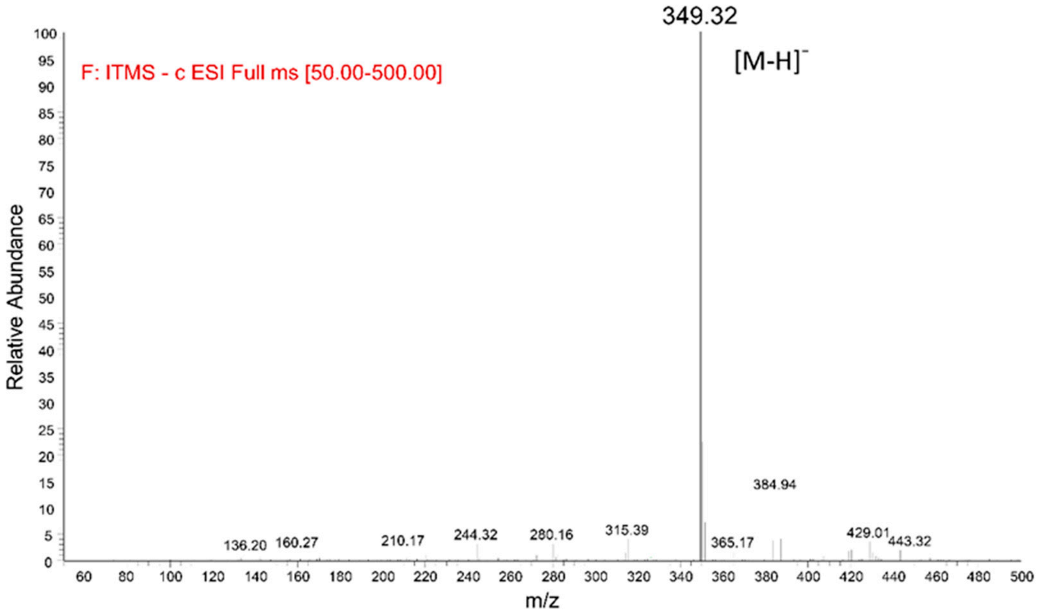

Figure S25. MS spectrum of NC 5.

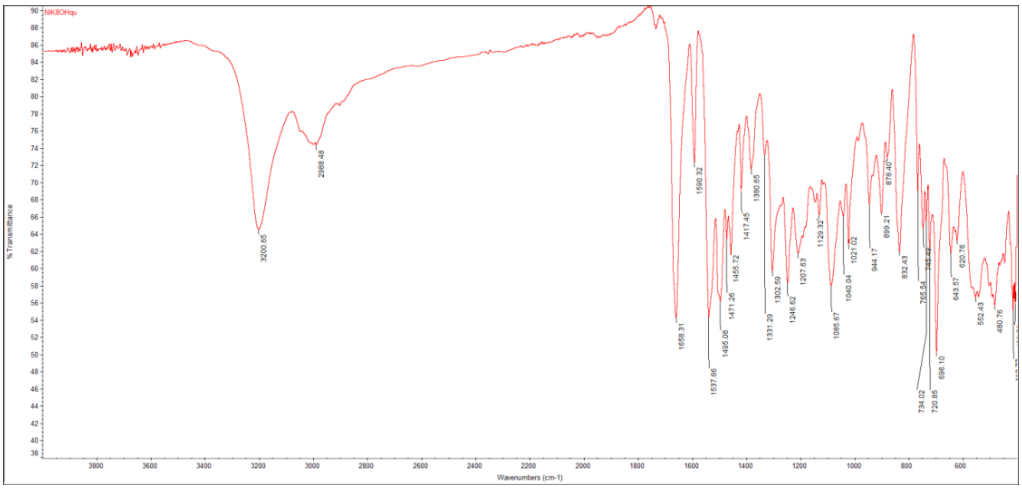

Figure S26. FTIR spectrum of NC 6**.**

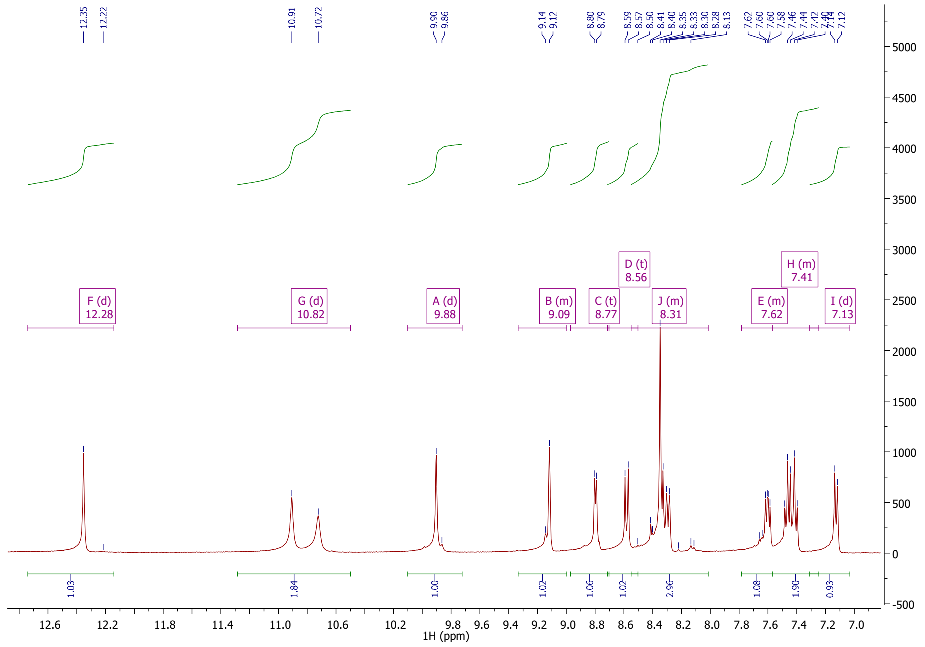

Figure S27. ^{1}H spectrum of NC 6.

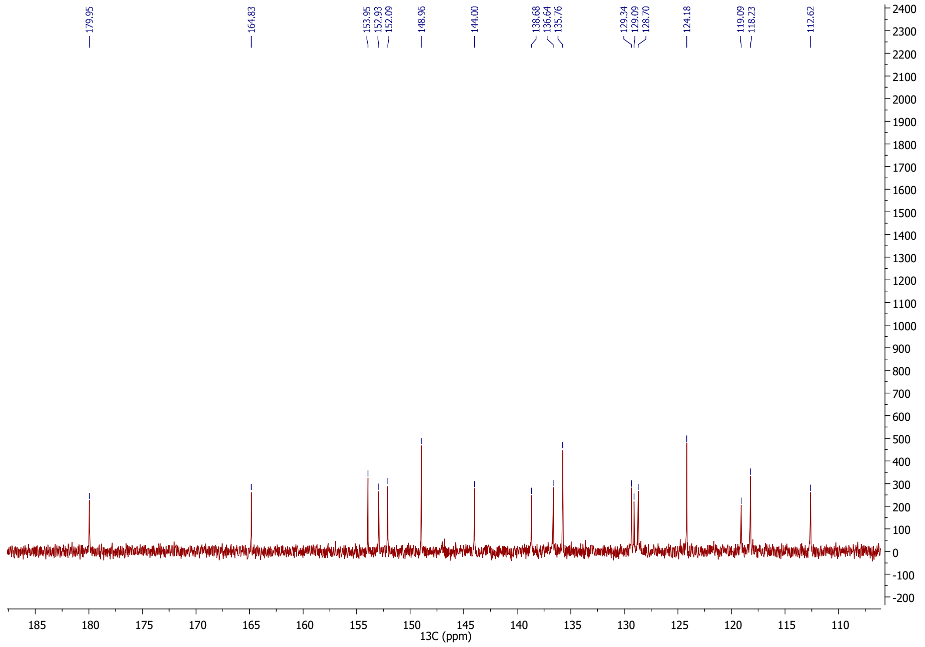

Figure S28. ^{13}C spectrum of NC 6.

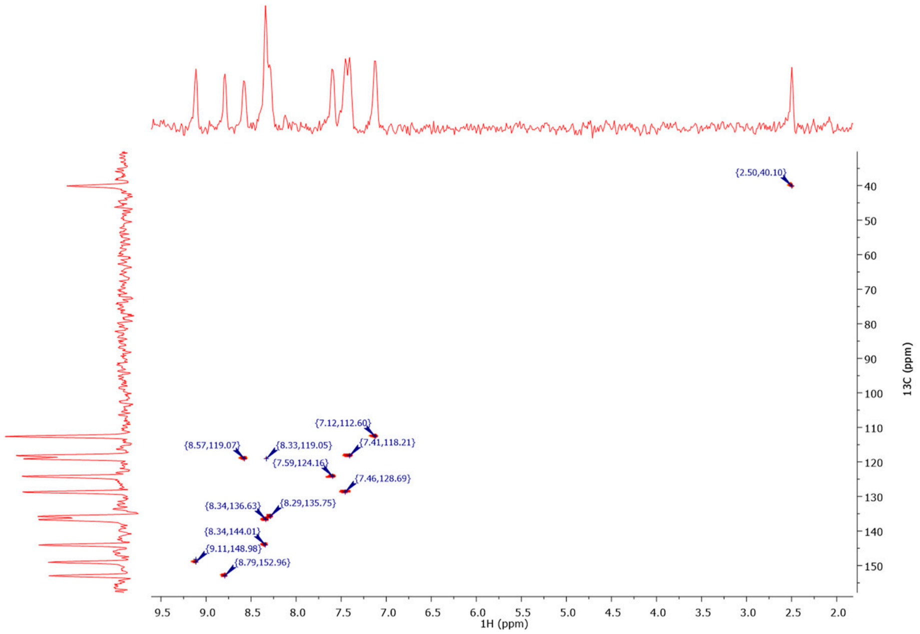

Figure S29. HSQC spectrum of NC 6**.**

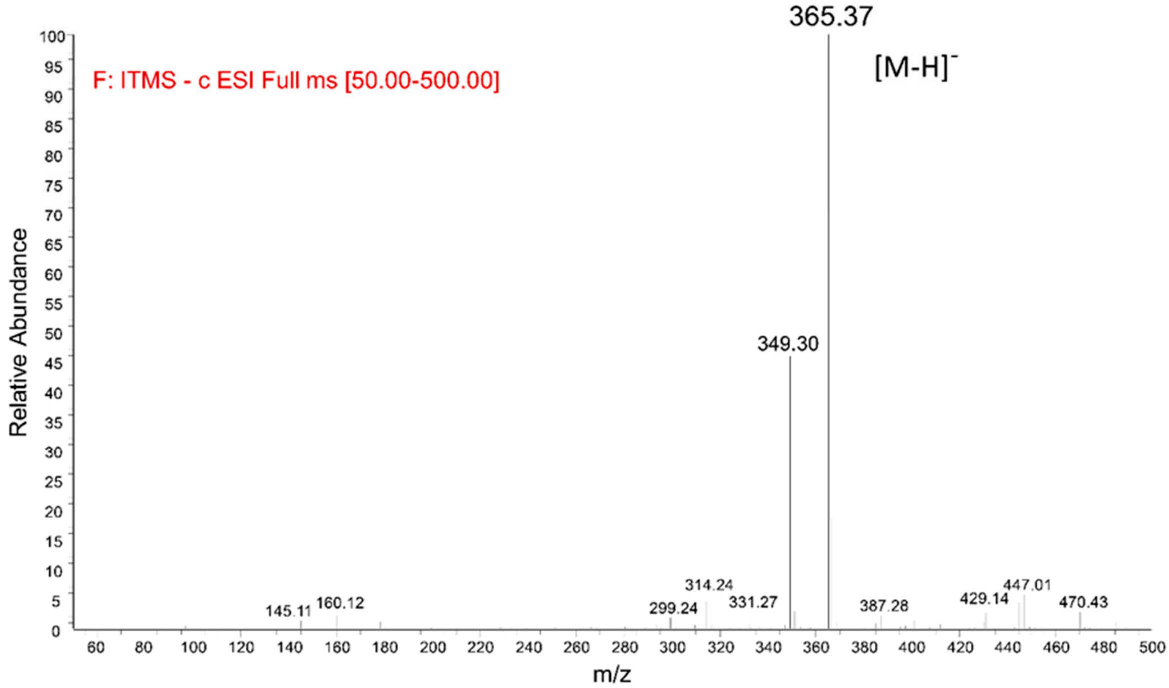

Figure S30. MS spectrum of NC 6.

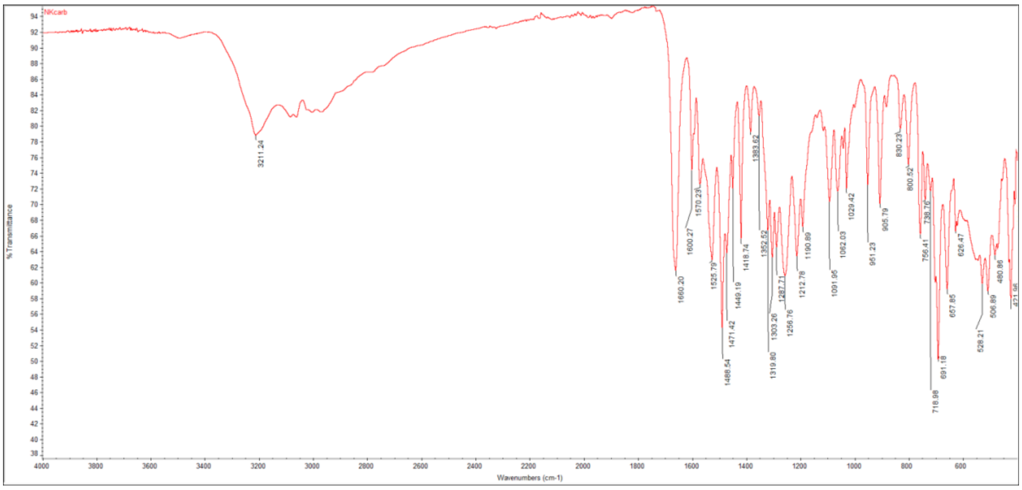

Figure S31. FTIR spectrum of NC 7.

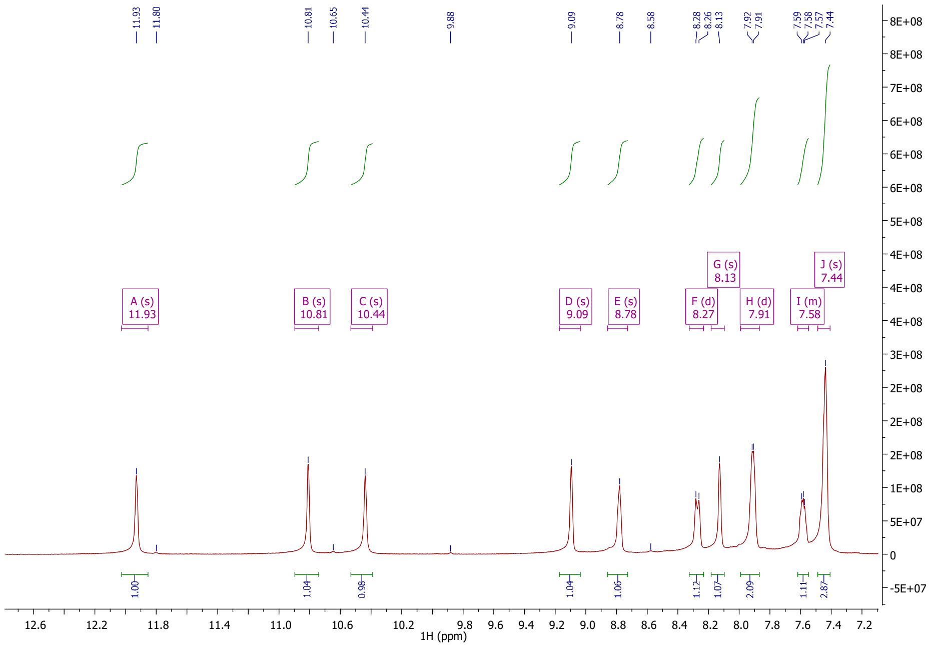

Figure S32. ^{1}H spectrum of NC 7.

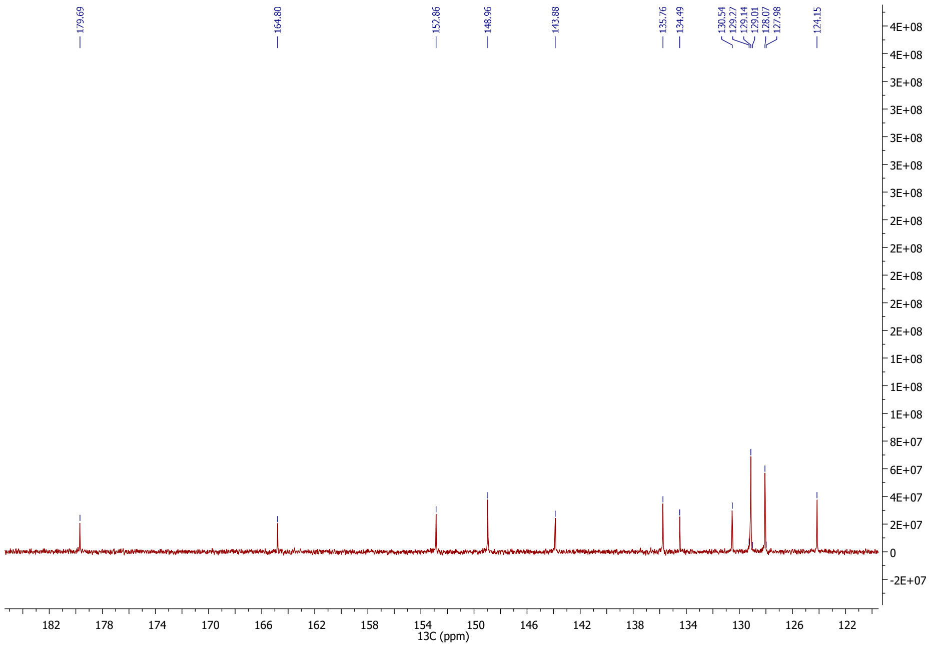

Figure S33. ^{13}C spectrum of NC 7.

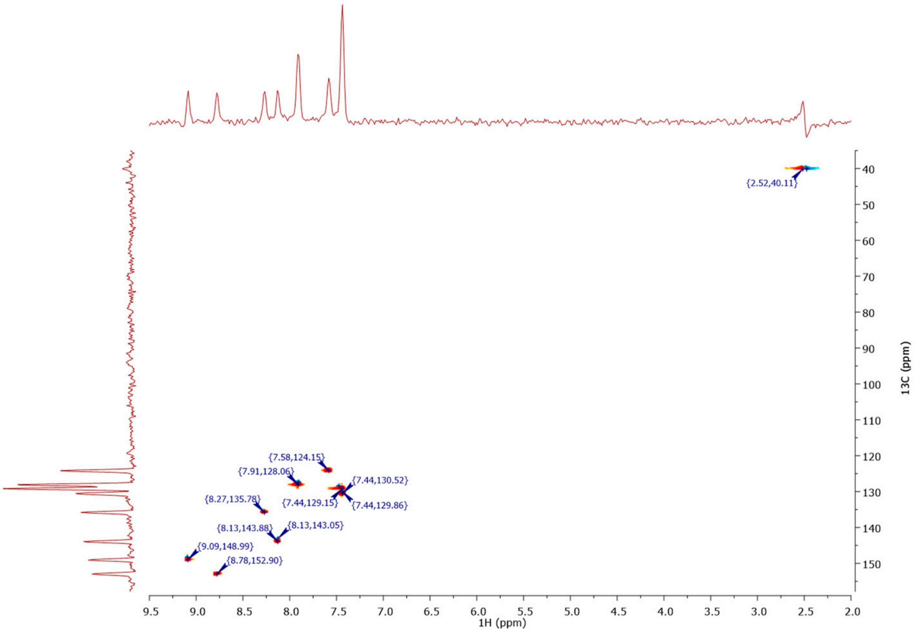

Figure S34. HSQC spectrum of NC 7.

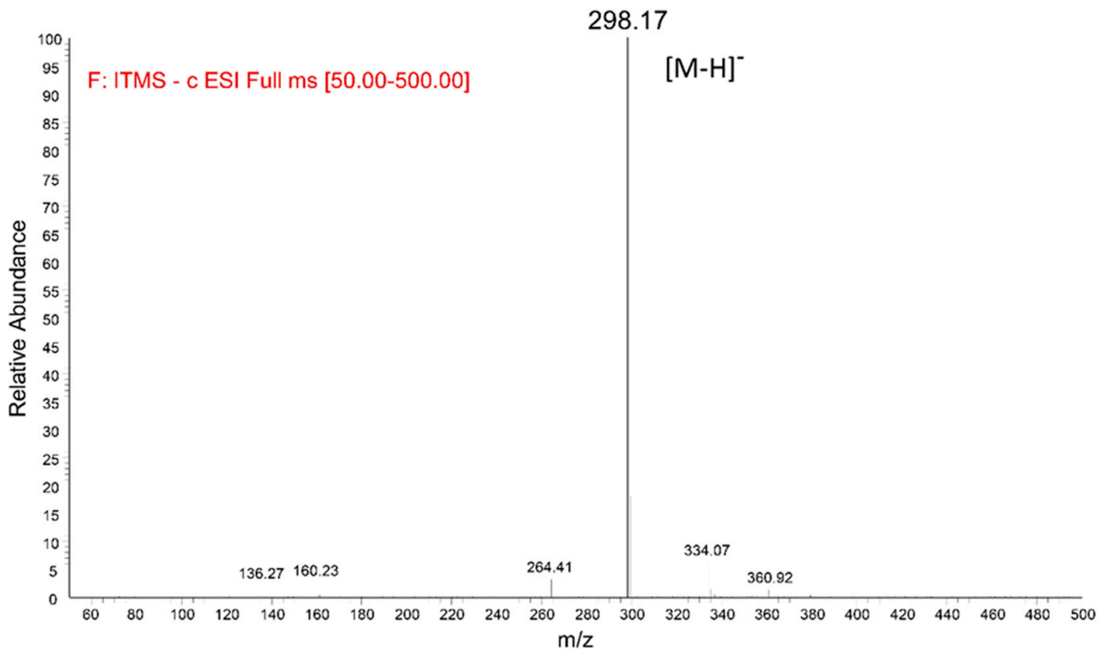

Figure S35. MS spectrum of NC 7.

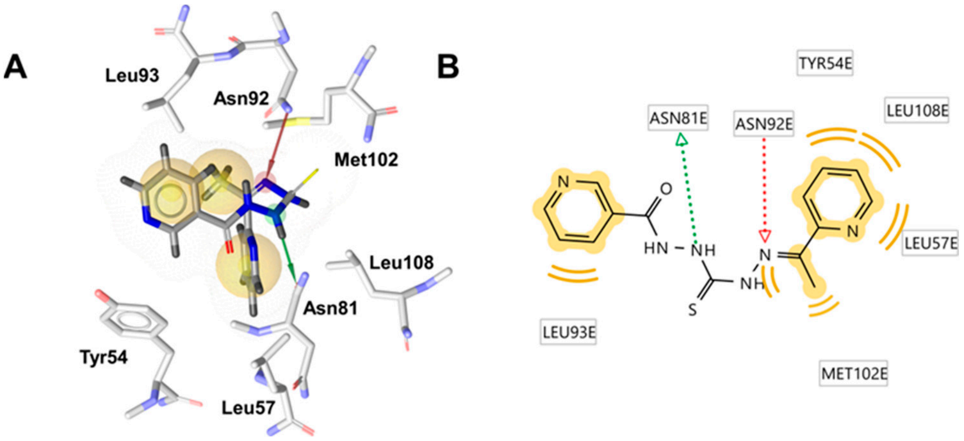

Figure S36. A) Binding mode of NC 3 within the active site of Streptogramin A acetyltransferase (VatD) (1KHR). The pharmacophores of the ligand are denoted by yellow spheres for hydrophobic interactions, green arrow for HBD interactions, and red arrow for HBA interactions with the specified amino acid residues; B) Corresponding 2D ligand interaction diagram (LID).

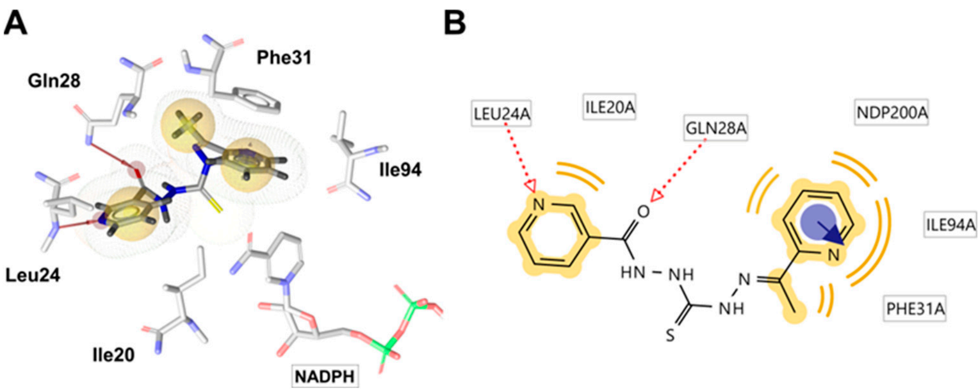

Figure S37. A) Binding mode of NC 3 into the active site of DHFR (1DG5). The pharmacophores of the ligand are labeled with yellow spheres for hydrophobic interactions, blue circles for π-π stacking, and green arrow for HBD interactions with the specified amino acid residues; B) Corresponding 2D ligand interaction diagram (LID).
